# Mass spectrometry imaging discriminates glioblastoma tumor cell subpopulations and different microvascular formations based on their lipid profiles

**DOI:** 10.1038/s41598-022-22093-4

**Published:** 2022-10-12

**Authors:** Kelly C. O’Neill, Evangelos Liapis, Brent T. Harris, David S. Perlin, Claire L. Carter

**Affiliations:** 1 Center for Discovery and Innovation, Hackensack Meridian Health, 111 Ideation Way, Nutley, NJ 07110 USA; 2 Departments of Neurology and Pathology, Georgetown University Medical Center, Washington, D.C. 20007 USA; 3 Department of Medical Sciences, Hackensack Meridian School of Medicine, Nutley, NJ 07110 USA; 4 Department of Pathology, Hackensack Meridian School of Medicine, Nutley, NJ 07110 USA

**Keywords:** Cancer microenvironment, Bioanalytical chemistry, Glycolipids, Phospholipids, Sphingolipids

## Abstract

Glioblastoma is a prevalent malignant brain tumor and despite clinical intervention, tumor recurrence is frequent and usually fatal. Genomic investigations have provided a greater understanding of molecular heterogeneity in glioblastoma, yet there are still no curative treatments, and the prognosis has remained unchanged. The aggressive nature of glioblastoma is attributed to the heterogeneity in tumor cell subpopulations and aberrant microvascular proliferation. Ganglioside-directed immunotherapy and membrane lipid therapy have shown efficacy in the treatment of glioblastoma. To truly harness these novel therapeutics and develop a regimen that improves clinical outcome, a greater understanding of the altered lipidomic profiles within the glioblastoma tumor microenvironment is urgently needed. In this work, high resolution mass spectrometry imaging was utilized to investigate lipid heterogeneity in human glioblastoma samples. Data presented offers the first insight into the histology-specific accumulation of lipids involved in cell metabolism and signaling. Cardiolipins, phosphatidylinositol, ceramide-1-phosphate, and gangliosides, including the glioblastoma stem cell marker, GD3, were shown to differentially accumulate in tumor and endothelial cell subpopulations. Conversely, a reduction in sphingomyelins and sulfatides were detected in tumor cell regions. Cellular accumulation for each lipid class was dependent upon their fatty acid residue composition, highlighting the importance of understanding lipid structure–function relationships. Discriminating ions were identified and correlated to histopathology and Ki67 proliferation index. These results identified multiple lipids within the glioblastoma microenvironment that warrant further investigation for the development of predictive biomarkers and lipid-based therapeutics.

## Introduction

Glioblastoma is the most common type of primary malignant brain tumor in adults and is classified by the World Health Organization as a Grade 4 neoplasm^1^. Despite surgical and therapeutic intervention, the prognosis for glioblastoma is bleak, with a median overall survival of 12–15 months after diagnosis^2^. The failure of therapeutic intervention is attributed to the intratumor heterogeneity in glioblastoma. Glioblastomas are difficult to fully resect and develop resistance to current therapeutic regimens, leading to high rates of recurrence, with limited treatment options^3^. Elucidating glioblastoma cellular heterogeneity within the tumor microenvironment (TME) is essential to identifying pathways that drive tumor progression for the development of curative therapeutics.

The Cancer Genome Atlas and two seminal publications using transcriptional studies laid the foundation for glioblastoma subtypes based on molecular profiles^4,5^. These studies identified the cellular composition of glioblastoma as either proneural, neural, classical, or mesenchymal. These subtypes were initially believed to contribute to glioblastoma heterogeneity between patients, but single cell RNA-seq demonstrated that all four were found within a single glioblastoma tumor^6^. The same study also identified a plasticity in cancer stem cell markers and reported a stemness gradient. These findings have been investigated in more detail recently, solidifying the notion that both tumor cells and glioblastoma stem cells (GSC) are not static and intratumoral plasticity results in diverse spatial and temporal heterogeneity that is shaped by the TME^7,8^. Despite these advancements in genomics and a greater understanding of the molecular heterogeneity within the glioblastoma TME, there are still no curative treatment options, and the prognosis is unchanged^2,3^.

Non-tumor cells within the microenvironment also contribute to glioblastoma progression. The perivascular niche (PVN) and the aberrant microvascular proliferation (MVP) that is a characteristic feature of glioblastoma helps drive the invasive nature of this tumor^9,10^. Studies have demonstrated that the perivascular niche maintains the GSC phenotype and contributes strongly to therapeutic resistance^11^. Despite the known roles of the PVN in the invasive and aggressive nature of glioblastoma, their phenotypes in relation to function remain ill-defined. The PVN has been categorized into subtypes based on their microvascular formations and include microvascular sprouting, vascular cluster, vascular garland and glomeruloid vascular proliferation^12^. These different vascular subtypes have been shown to correlate with prognosis^13^. Given the known involvement of the PVN and the failure of anti-angiogenic therapies in clinic; a greater understanding of the phenotype of the endothelial cells that comprise the TME is needed^14^.

Lipids are key regulatory molecules that have been intricately linked to cell fate through direct and indirect signaling mechanisms with roles in proliferation^15^, differentiation^16^ and apoptosis^17^. It is thus unsurprising that altered lipid metabolism and cellular composition have been linked to cancer cell growth^18^, aggression^19^, metastasis^20^ and resistance to therapy^21^. Previous research has shown there are increased levels of free and lipid-incorporated long chain polyunsaturated fatty acids (PUFA) in GSCs that support oncogenic EGFR signaling^22^. The sphingolipid rheostat, a determinant of cell fate, is also dysregulated in glioblastoma, leading to a reduction in growth arrest pathways and an increase in proliferation pathways^23^. Many of these dysregulated lipid pathways are considered therapeutic targets^21^.

Mass spectrometry imaging (MSI) has been used previously to analyze the spatial distribution of lipids in preclinical and clinical glioblastoma samples^24–26^. These studies have focused on the classification of glioblastoma from lower grade gliomas based on lipid profiles^24^ and changes between different morphological tissue regions such as tumor vs. stroma^25,27^. In the current study, high resolution MALDI Fourier transform ion cyclotron resonance MSI was used to determine the first intratumoral lipid heterogeneity within cancer cell subpopulations and the different subtypes of microvascular formations in human glioblastoma samples. Ceramide-1-phosphates, gangliosides, cardiolipins and phosphatidylinositiols were able to differentiate histologically similar regions of high tumor cell density, regions of pseudopalisading tumor cells and the different formations of MVPs. A loss in sphingomyelin, a lipid that is abundant in normal brain, was observed in all low–high tumor cell regions. Lastly, sulfatides were able to differentiate low-moderate tumor cell regions from high tumor cell regions and regions of pseudopalisading tumor cells.

## Results and discussion

Histological analysis, mass spectrometry imaging and Ki67 immunostaining were carried out on five human glioblastoma samples. The deadly nature of glioblastoma and the timing of resection and banking of these samples meant that each patient underwent different treatment regimens. These included radiation alone or in combination with temozolomide, + /− bevacizumab or pembrolizumab, and the anti-seizure medications Kepra/Vimpat. Due to the diversity of the treatment regimens for each patient and the possible impact these could have on lipid profiles, initial investigations focused on intratumor heterogeneity within each sample. Slightly deeper sections were then taken for repeat MSI analysis and Ki67 staining to correlate lipid profiles with proliferation index. Discriminant analysis of these samples enabled the identification of several lipids that accumulated within high tumor cell regions with high Ki67 labeling across all samples analyzed, regardless of pathology or treatment. These results are discussed in detail in the following sections.

### Histological and molecular intra- and intertumor heterogeneity

**Figure 1:**
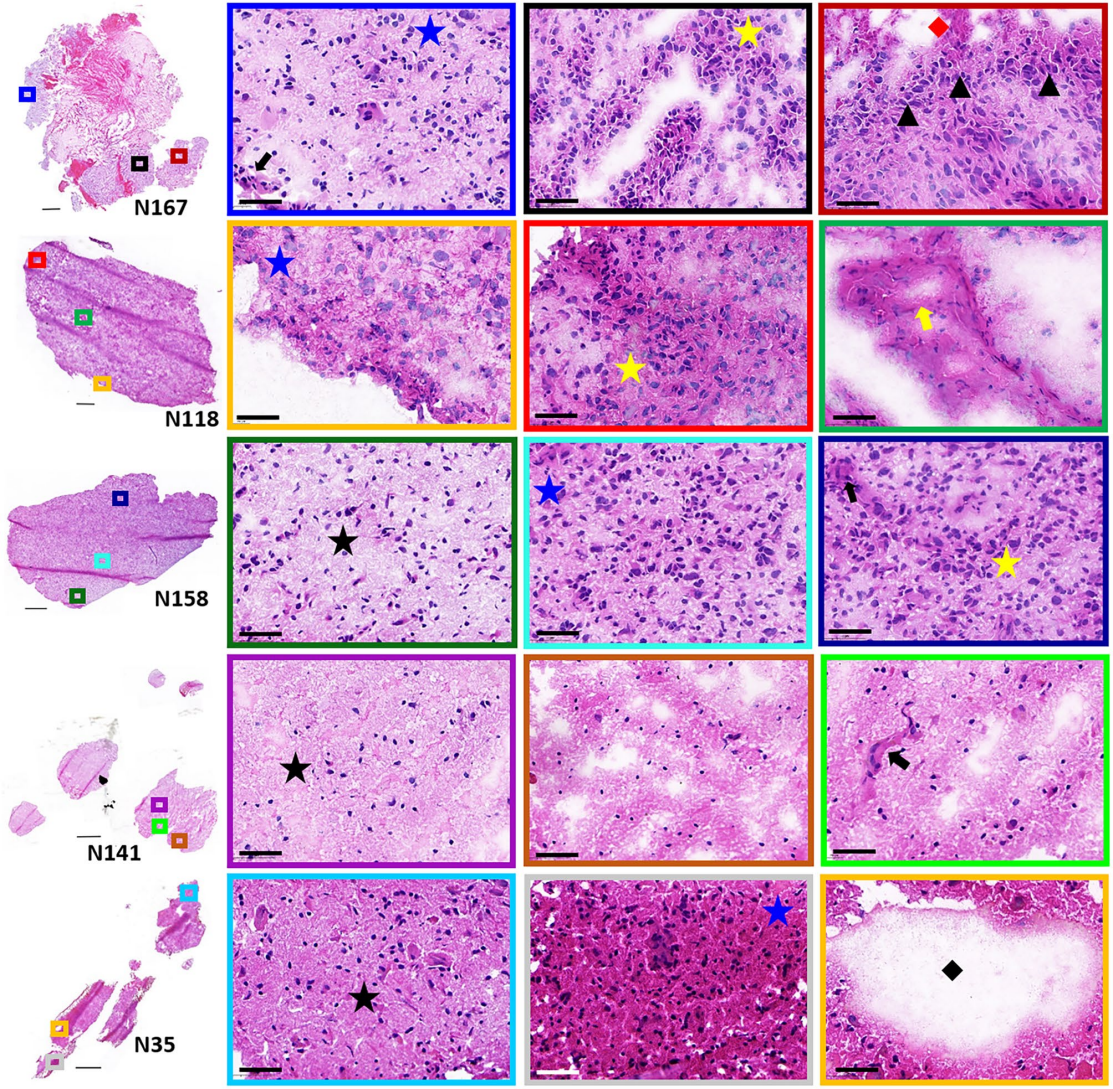
H&E stained sections of patient glioblastoma samples. Higher magnification of representative regions are shown in the colored boxes. Histologic features include low (black star), moderate (blue star) and high (yellow star) tumor cell densities, pseudopalisading tumor cells (black arrow-head), microvascular sprouting (black arrow) and vascular garland formations (yellow arrow), necrosis (red diamond) and acellular fluid-filled regions (black diamond). Scale bars are 50 µm.

Histological interpretation of each patient glioblastoma samples used in this study was carried out by a board-certified neuropathologist. Glioblastomas present with diverse histological features that include hypercellularity; atypia; pleomorphic nuclei; pseudopalisades surrounding necrosis and different formations of MVP. These histological features are shown in the patient samples presented in Fig. 1, Supplementary Fig. S1–5. The section taken from sample N167 displayed the most diverse histology. This section contained large areas of hemorrhage surrounded by necrosis, regions of moderate and high tumor cell density, pseudopalisading tumor cells and MVPs encompassing vascular garland structures and microvascular sprouting. Histological observations of N118 identified moderate tumor cell density and minimal pleomorphic nuclei with a high-density fibrillary background. Regions of MVP were identified throughout this section that spanned varying pathological formations (Supplementary Fig. S2). Glioblastoma sample N158 displayed a heterogeneous histological gradient across the tissue section. The regions along the bottom and right side of this section had a lower tumor cell density composed of smaller cells and reactive microglia. Tumor cell density increased from the middle to the top of this section, which contained high density tumor cells with mildly pleomorphic nuclei and regions of microvascular sprouting. The section from sample N141 displayed histological parenchyma that more closely resembled normal brain. This section contained a higher number of reactive astrocytes and few tumor cell nuclei. (Supplementary Fig. S4). Finally sample N35 consisted of a mild tumor cell infiltrate compared to samples N167 and N118 and contained several acellular fluid-filled pockets (Supplementary Fig. S5).

Due to the complexity and high‐dimensional nature of MSI data and to remove any potential bias in data processing, the datasets were initially processed using probabilistic latent semantic analysis^28^. This unsupervised multivariate classification method automatically extracts the underlying trends in the data based on the ions detected. The score images presented in Supplementary Fig. S6 display the main spatial features based on the component mass spectra, which highlight the intra- and intertumor heterogeneity based on the spatial distribution and relative intensity of lipid ions. Single and merged ion images were created based on these results and are discussed in detail in the forthcoming sections.

### Lipidomic heterogeneity within the glioblastoma microenvironment

#### Cardiolipins- mitochondrial specific lipids

**Figure 2:**
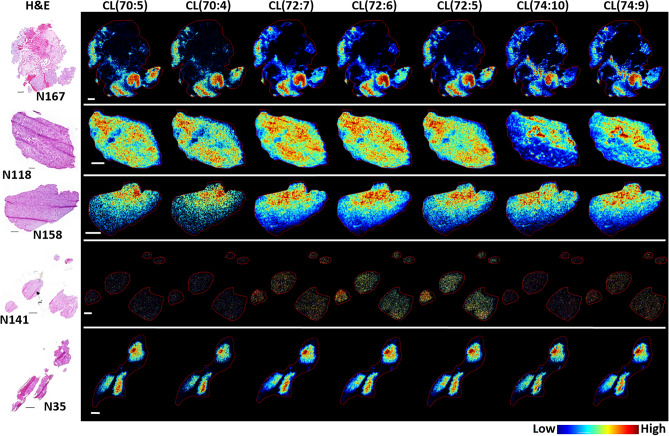
Representative MS images of several cardiolipin (CL) species. CL(70:5), (70:4), (72:7), (72:6), (72:5), (74:10) and (74:9) correspond to *m/z* 1425.984, 1427.997, 1449.978, 1451.995, 1454.011, 1471.966 and 1473.981, respectively. Images were acquired at 30 µm pixel resolution from 5 µm thick sections; the H&E stained section of each sample is shown in the left panel. Scale bars are 1 mm.

Cardiolipins (CLs) are a mitochondrial specific phospholipid that are predominantly located in the inner mitochondrial membrane (IMM); the composition of their fatty acid residues play important roles in mitochondrial structure and function^29^. Representative MS images of CL distribution are presented in Fig. 2. In samples N167, N118 and N158, CLs were predominantly detected in regions of high tumor cell density, pseudopalisading tumor cells, which are an actively migrating cell population^30^, and the MVP formations characterized as microvascular sprouting and vascular clusters. The accumulation of several CLs in samples N167 and N118 demonstrated histological structure-based heterogeneity that was dependent upon the carbon number and levels of unsaturation in their fatty acid residues. This can be observed by comparing the distribution of CL(70:4) through to CL(74:10). A decrease in the detection of CLs was observed in the regions containing pseudopalisading tumor cells in sample N167 as the carbon number and levels of unsaturation increased. Conversely, the signal intensity of longer chain CLs increased in the microvascular formations surrounding necrosis in N167, and in distinct regions of tumor cells and vascular clusters in sample N118. A gradient of no signal to high signal was observed for all CLs in sample N158; correlating with low-to-high tumor burden in this sample. Similarly, all CLs were detected at or near noise level in sample N141, which contained few tumor cells and more closely resembled normal brain parenchyma. In sample N35, CLs were detected with highest intensity in regions surrounding the acellular fluid filled areas. The histology-based distribution and corresponding signal intensity for all CLs detected are presented in Supplementary Table S1.

Prior to this study, much of the information on CL dysregulation in glioblastoma has been derived from in vitro investigations and thus a single cell phenotype. Treatment of the glioblastoma cell line GL15 with palmitate or bromopyruvate resulted in a loss or degradation of CL species, respectively^31,32^. This destabilized CL interaction with cytochrome *c* leading to its release and the induction of the apoptosis cascade in these cells. While there is limited information on CLs in glioblastoma, several recent studies have demonstrated CL alterations and/or overexpression of the CL acetyltransferase, tafazzin, in prostate cancer^33^, thyroid cancer^34^, glioma^35^, pancreatic cancer stem cells^36^ and cervical cancer^37^. Upregulation of the CL modifying enzyme, lysocardiolipin acyltransferase (LYCAT) was also recently detected in non-small cell lung cancer and knockdown of LYCAT reduced cell proliferation, migration, invasion, and thus tumor size and burden in vitro and in vivo^38^. Understanding the functional relevance of CL composition in relation to their accumulation in the different tumor cell and endothelial cell populations in this study warrants further investigation as they offer novel therapeutic targets for the treatment of glioblastoma.

#### Phosphatidylinositols

**Figure 3:**
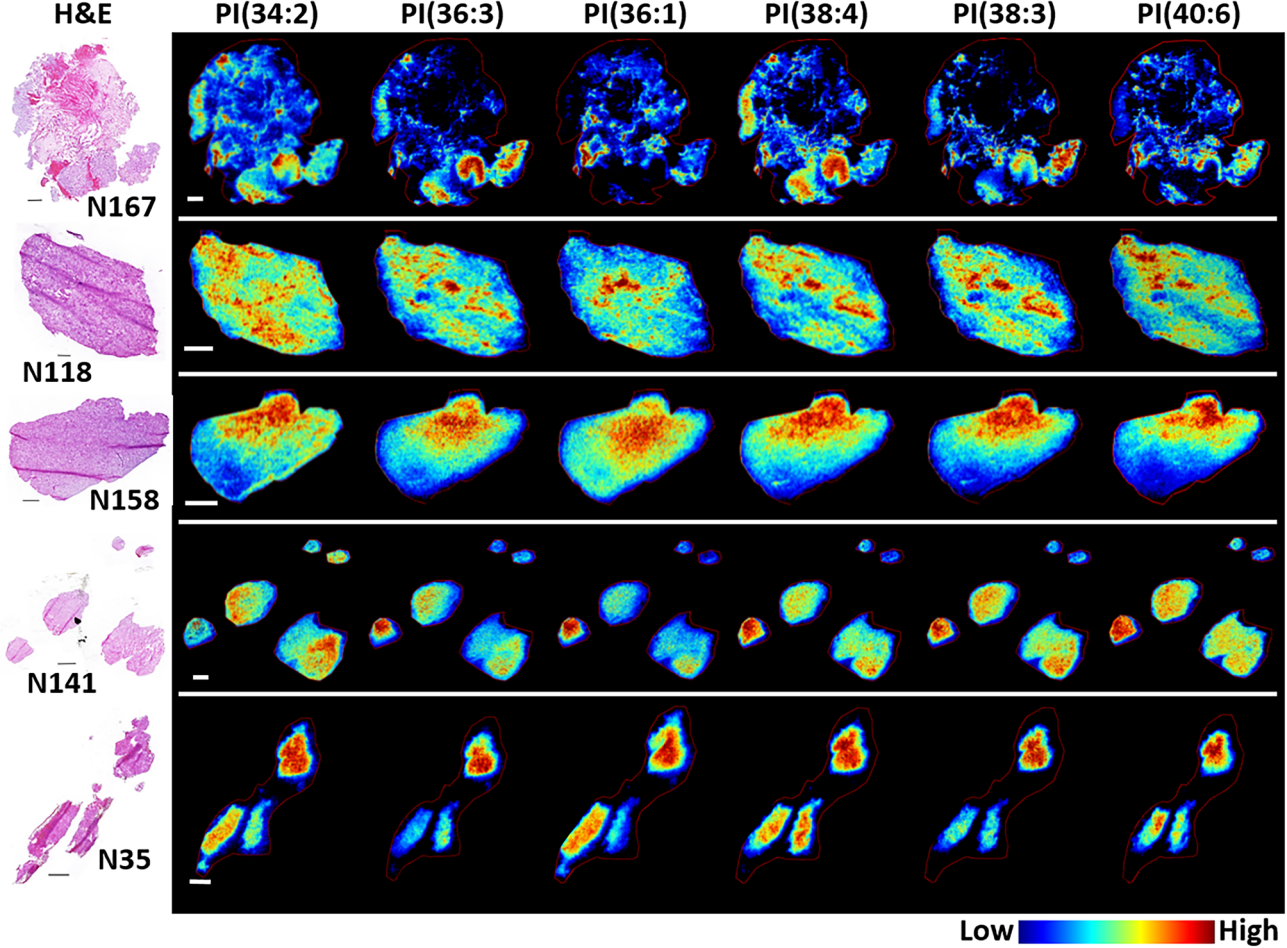
Representative MS images of several phosphatidylinositol (PI) species. PI(34:2), (36:3), (36:1), (38:4), (38:3) and (40:6) correspond to *m/z* 833.521, 859.535, 863.564, 885.550, 887.562 and 909.552, respectively. Images were acquired at 30 µm pixel resolution from 5 µm thick sections; the H&E stained section of each sample is shown in the left panel. Scale bars are 1 mm.

Phosphatidylinositols (PIs) are important cell signaling lipids and precursors for the PI3K pathway, which is dysregulated in many cancers^39^. A total of 16 PIs were detected and descriptions of the pathological regions in which PIs were localized are available in Supplementary Table S2; representative PI images are displayed in Fig. 3. PI species were differentially distributed within the glioblastoma TME and this heterogeneity was again dependent on their fatty acid composition. In sample N167, the localization and accumulation of PIs in the cancer cell regions demonstrated a differential distribution within histologically similar neighboring cells from the same region and across tumor cell regions in different areas of this section. This can be observed by comparing the spatial distribution and signal intensity of PI(34:2), (36:3), (38:4), (38:3) and (40:6) to one another. For example, PI(38:4) was highly localized to the moderately dense tumor cell regions near the bottom, whereas PI(38:3) was most abundant in the bottom right region consisting of higher tumor cell density, pseudopalisading tumor cells, and MVP. The distribution and accumulation of PIs in sample N118 demonstrated similar trends to those observed for CLs, in that relatively high signal intensity was detected throughout the tumor cell regions, with the highest signals differentially distributed across distinct pathological regions of MVPs. A gradient in the signal intensity detected for all PI lipid species was again evident in sample N158 that correlated with low-to-high tumor cell density. In samples N141 and N35, PI species were detected with different abundances in each biopsy sample. Unlike CLs, however, PIs were detected with high signal intensity in sample N141. These were predominant in the two larger biopsy samples and localized to areas that contained MVPs. In sample N35, PIs demonstrated a more homogeneous distribution within each biopsy sample with the highest signal intensity detected throughout the top biopsy section, which contained a large acellular fluid filled region and vascular clusters. A complete description of all PIs detected and their histology-based distribution in all five samples is presented in Supplementary Table S2.

PIs play central roles in cell signaling and regulation that is dictated by both the composition of their fatty acid residues and phosphorylation at the 3’, 4’ and 5’ position of their inositol head group, which gives rise to the second messengers, phosphatidylinositol phosphates (PIPs)^40^. The enzymes involved in the regulation of PIPs and thus PIP signaling are some of the most well-known and studied in cancer^41^. Phosphoinositide-3-kinase (PI3K), produces PIP3, which activates the AKT and the mammalian target of rapamycin (mTOR) signaling pathways^42^. The PI3K/AKT/mTOR pathway is upregulated in most human cancers and drives cell survival, proliferation and invasion^41,42^. Conversely, phosphatase and tensin homolog (PTEN), the enzyme responsible for dephosphorylation of PIP3 and known as a tumor suppressor, is silenced in many human cancers^41^. The impact of PI fatty acid composition in regulation of these pathways during glioblastoma development and progression is unknown. Recent studies, however, have shown that alterations to PI fatty acyl chain composition altered AKT localization and downstream signaling in a prostate cancer model^43^. Fatty acyl chain remodeling of PIs was also shown during the analysis of primary breast cancer tissue^44^. This study identified PI(38:3) as a hallmark of invasive cancer cells that was associated with higher incidence of lymph node metastasis^44^. In the current study, this lipid was also highly localized to regions containing actively migrating cell populations, histologically characterized as pseudopalisading tumor cells^30^. Understanding the functional relevance of PI(38:3) in these migrating glioblastoma cell populations is an important next step of research. Additionally, the PTEN/PI3K/AKT/mTOR pathway has been extensively studied in glioblastoma and a number of pathway inhibitors are in preclinical and clinical trials^45^. Targeting the downstream PIs that feed into this pathway may offer a novel therapeutic approach.

#### Sphingolipids

**Figure 4:**
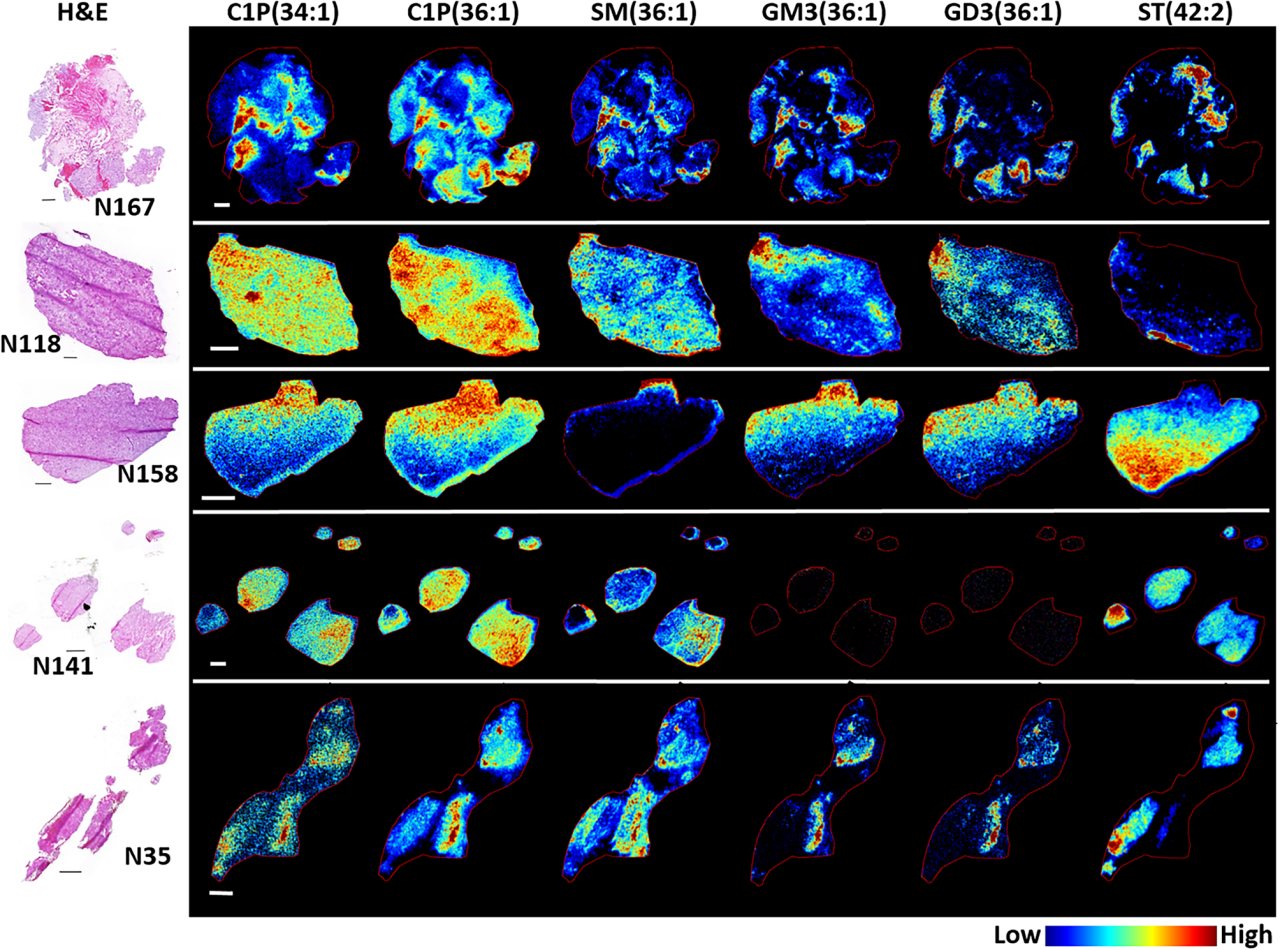
Representative MS images for several sphingolipid species. Sphingolipid subclasses include ceramide-1-phosphate (C1P), sphingomyelin (SM), mono- and di-sialodihexosylganglioside (GM3 and GD3 respectively) and sufatide (ST). C1P(34:1) and (36:1) correspond to *m/z* 616.471 and 644.502, respectively. SM(36:1) corresponds to *m/z* 715.578. GM3(36:1) and GD3(36:1) correspond to *m/z* 1179.741 and 1470.830, respectively. ST (42:2) corresponds to *m/z* 888.624. Images were acquired at 30 µm pixel resolution from 5 µm thick sections; the H&E stained section of each sample is shown in the left panel. Scale bars are 1 mm.

Sphingolipids are a diverse family of lipids involved in numerous cell signaling processes that regulate cell division, differentiation, migration and apoptosis. Over 40 different sphingolipids were detected, encompassing the subclasses of ceramide-1-phosphates (C1P), sphingomyelin (SM), sulfatides (ST) and gangliosides. A full list of the sphingolipids detected along with their structure-based histology-specific accumulation within each sample are presented in Supplementary Tables S3, S4, S5, S6 Representative images demonstrating the structure-based heterogeneous distribution for each subclass is shown in Fig. 4. With the exception of sulfatide, sphingolipids were predominantly localized to regions of necrosis (N167), acellular fluid filled regions (N35), and areas of MVPs (N167, N118 and N35). Few C1Ps and gangliosides were detected within the tumor cell regions and those that were shared similar carbon chain lengths and levels of saturation in their fatty acid structures, irrespective of their sub-class. This can be observed by comparing the spatial distribution of C1P(36:1), GM3(36:1) and GD3(36:1) to one another. All of these lipids were accumulated within the same regions of moderate-to-high tumor cell density in samples N167 and N158. C1P(36:1) was also highly accumulated in regions of MVP surrounding necrosis in samples N167 and the MVP formations of microvascular sprouting and vascular clusters in sample N118. Interestingly, in sample N118, C1P(34:1) was more highly accumulated in the vascular garland structure when compared to C1P(36:1).

C1Ps are part of the sphingolipid rheostat that determines cell fate, in which ceramide promotes cell growth arrest and death through multiple signaling pathways, whereas metabolism of Cer to sphingosine-1-phosphate (S1P) or phosphorylation to C1P promotes cell growth and proliferation^17,46^. C1P has also been shown to be a potent chemoattractant involved in the trafficking of immune and stem cell populations through mechanisms that are not fully understood^47^. While much research has focused on the roles of ceramide and S1P in cancer progression, less is known about C1P in cancer and there are no studies on its role in glioblastoma^17^. C1P and ceramide kinase (CerK), the only enzyme identified to date that phosphorylates Cer to form C1P, have been implicated in breast^48–51^, lung^50,52,53^ and pancreatic cancer^54,55^ cell growth, survival and dissemination. In breast and lung cancer, upregulation of CerK was shown to promote tumor cell survival and migration/invasion of cancer cells through activation of the PI3K/AKT, ERK1/2 and the Rho kinase signaling pathways^49,52^. CerK expression has also been associated with breast cancer aggression and recurrence, and thus patient prognosis^48,51^. Inhibition of CerK with NVP-231, reduced C1P levels and resulted in M phase growth arrest in breast and lung cancer cell lines and decreased migration of metastatic breast cancer cells^49,50^. In pancreatic cancer cells, C1P stimulated cell migration and invasion was shown to be dependent upon the PI3K, AKT1, mTOR1, MAPK, ERK1-2 and RhoA/ROCK signaling pathways^54,55^. C1P signaling in pancreatic cancer cells has been shown through both intracellular and extracellular signaling mechanisms. A recent study demonstrated pancreatic ductal adenocarcinoma secreted C1P-containing extracellular vesicles, which promoted motility and recruitment of pancreatic cancer stem cells, suggesting C1P-related signaling mechanisms facilitate pancreatic tumor progression^54^. The pro-survival mechanisms of S1P and C1P may explain why many S1P inhibitors have failed in clinical trials, as C1P may be an alternative and compensatory pro-survival signaling molecule driving tumor progression. Similar to the distribution of CLs and PIs, a number of C1Ps were detected and differentially distributed within different cellular regions and pathological presentations of MVPs. Very little is known about the cellular phenotypes that constitute these pathologies in glioblastoma. Several studies, however, have demonstrated CerK activity and C1P formation was essential for both microvascular proliferation and the recruitment of endothelial progenitor cells and thus is pro-angiogenic^56^. Inhibition of C1P may offer a potential therapy targeting both select tumor cell populations and the aberrant microvascular proliferation observed in glioblastoma.

Gangliosides are a class of glycosphingolipids composed of a ceramide moiety linked to a glycan head group that contains varying numbers of sialic acid residues. The disialogangliosides with 2 and 3 glycosyl groups, GD2 and GD3, have been shown to be highly abundant in numerous brain tumors^57,58^. GD2 expression in pediatric and adult brain tumors has been extensively studied and the anti-GD2 monoclonal antibodies, dinutuximab and naxitamab, are FDA approved for the treatment of neuroblastoma^59,60^. Anti-GD2 CAR T cells have also recently demonstrated efficacy in the treatment of several brain tumors and are under investigation in clinical trials (NCT02761915, NCT03423992, NCT04099797). Of particular interest in the current study is the detection and distribution of GD3(36:1) within sub-populations of glioblastoma tumor cells along with its absence in sample N141. Mass spectrometry analysis of bulk homogenized brain tissue demonstrated GD3(36:1) was the predominant ganglioside in glioblastoma patient samples^61,62^. GD3 and GD3 synthase have also been identified as a biomarker for glioblastoma stem cells and treatment with a GD3 antibody suppressed tumor growth in vitro and in vivo^63,64^. GD3 involvement in pre/pro-tumorigenic events in the microenvironment away from the tumor margin was suggested following an in vivo study that detected GD3, NG2 and loss of PTEN in cells that were yet to undergo histologically identifiable neoplastic transformation^64^.

Sulfatide and sphingomyelin are highly distributed to normal brain parenchyma where they are major components of the myelin sheath^65,66^. In the cellular regions of samples N167, N118 and N158, STs were predominately detected and localized in regions consisting of lower tumor cell density and a more normal neuropil composition. Minimal-to-no signal was detected for this lipid class in areas of moderate-to-high tumor cell density, as evidenced by the example data presented for this lipid class in Fig. 4. The distribution of sulfatides in N158 also had an inverse intensity gradient compared to the aforementioned lipids detected in this sample, where high signal intensity for all STs were detected in the lower half of the section, which consisted of few tumor cells and relatively normal brain parenchyma. Interestingly, despite their shared accumulation within the myelin sheath and other components of the normal brain parenchyma, SMs mostly demonstrated a differential distribution to those detected for STs. SMs were predominantly localized to regions of necrosis and MVPs in all samples. While the role of SM in MVPs remain unknown, loss or reduction of SM in glioblastoma cells has been reported^66^. Over expression of the SM metabolizing enzyme, acid sphingomyelinase (ASM), was also shown to be associated with shorter survival in patients^67,68^. Therapeutics targeting sphingolipid metabolism have shown efficacy in the treatment of glioblastoma during preclinical studies. Treatment of glioblastoma cells in vitro and in vivo with the non-toxic membrane lipid therapy drug, 2-hydroxyoleic acid (2OHOA), recovered SM cell composition^67,69^ and downregulated the MAPK/PI3K/AKT pathways, leading to cell death and reduced tumor burden^69^. The safety of 2OHOA is currently being tested when used alongside conventional glioblastoma therapies (NCT03867123 and NCT04250922). A recent study has also provided evidence linking SM metabolism with EGFR oncogenic receptor signaling on the plasma membrane of glioblastoma cells. By inhibiting ASM activity with the antidepressant drug, fluoxetine, the authors demonstrated the accumulation of SMs resulted in a loss of EGFR receptors from membrane lipid raft domains and the killing of glioblastoma cells via activation of lysosomal stress. These studies highlight the importance of the evolving field of membrane lipid therapy and the significance of this study in spatially identifying lipidomic alterations for the identification of lipid pathway therapeutic targets.

**Figure 5:**
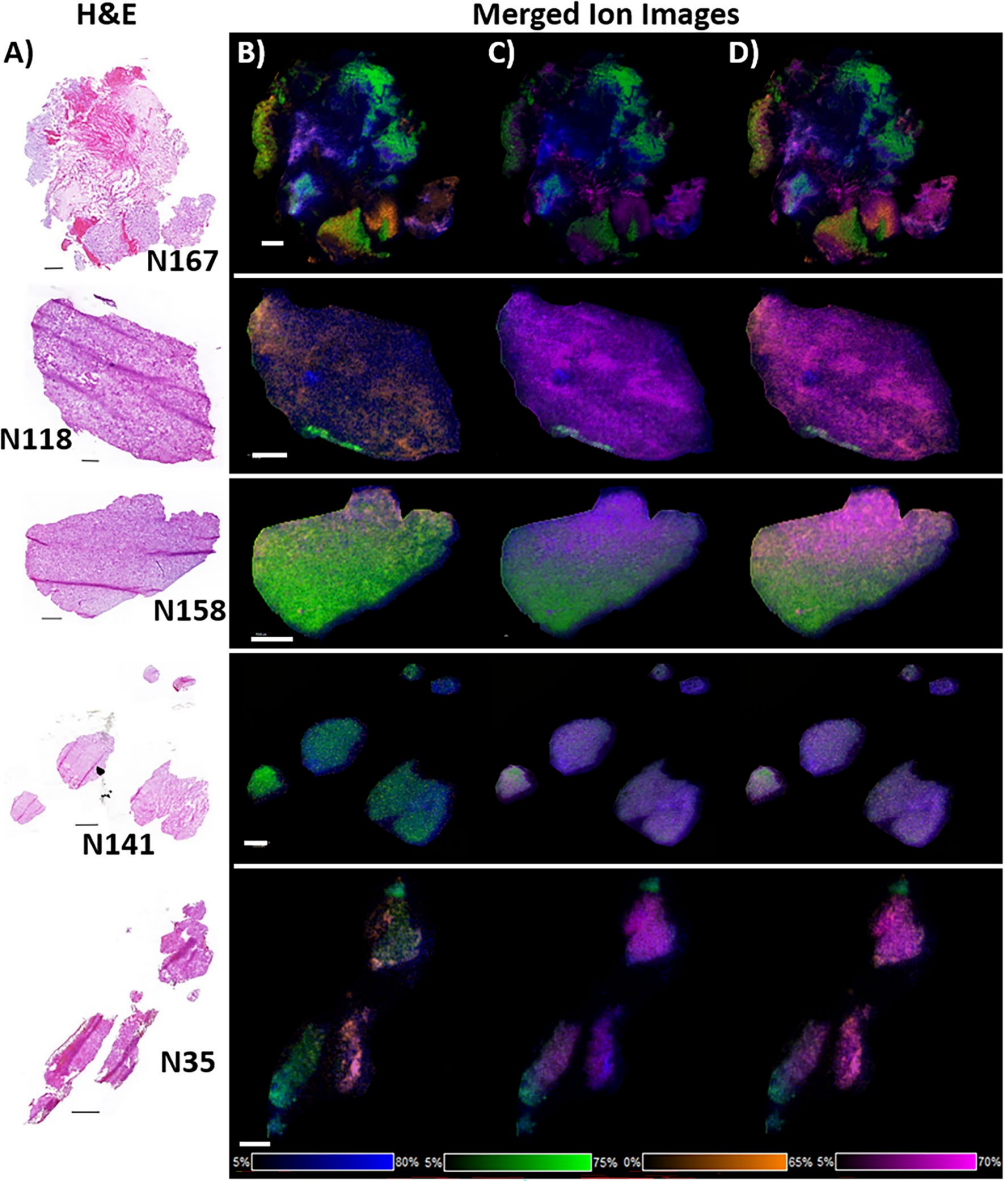
Merged ion images displaying the spatial distribution of multiple lipid classes**.** (**A**) H&E stained section of each sample is shown in the left panel. (**B**) Merged MS images of GD3(36:1) at *m/*z 1470.830 in displayed using orange, C1P(34:1) at *m/z* 616.471 in blue, and ST(42:2) at *m/z* 888.624 in green. (**C**) Merged MS images of PI(38:3) at *m/*z 887.562 is shown in pink, C1P(34:1) at *m/z* 616.471 in blue and ST(42:2) at *m/z* 888.624 in green. (**D**) Merged MS images of GD3(36:1) in orange, PI(38:3) in pink, C1P(34:1) in blue and ST(42:2) in green. Images were acquired at 30 µm pixel resolution from 5 µm thick sections. Scale bars are 1 mm.

To further highlight the histology-based lipid complexity in the glioblastoma TME in patient samples, several merged ion images were generated, displaying the spatial distribution of multiple lipids in each section (Fig. 5). In all MS images, ST(42:2) was selected as a marker of more normal brain parenchyma (green channel) and C1P(34:1) as a marker for both microvascular sprouting and the vascular garland structures (blue channel). These were merged with the GSC marker GD3(36:1) (orange channel) in Fig. 5B and the high-tumor cell/migratory pseudopalisading tumor cell region marker, PI(38:3) (pink channel), in Fig. 5C. All four lipid ions are presented together in Fig. 5D.

A full list of species detected for the lipid classes discussed, along with their *m/z*, mass deviation, ion formation and chemical formula, are presented in Supplementary Tables S7-S12.

### Discriminant analysis

**Figure 6:**
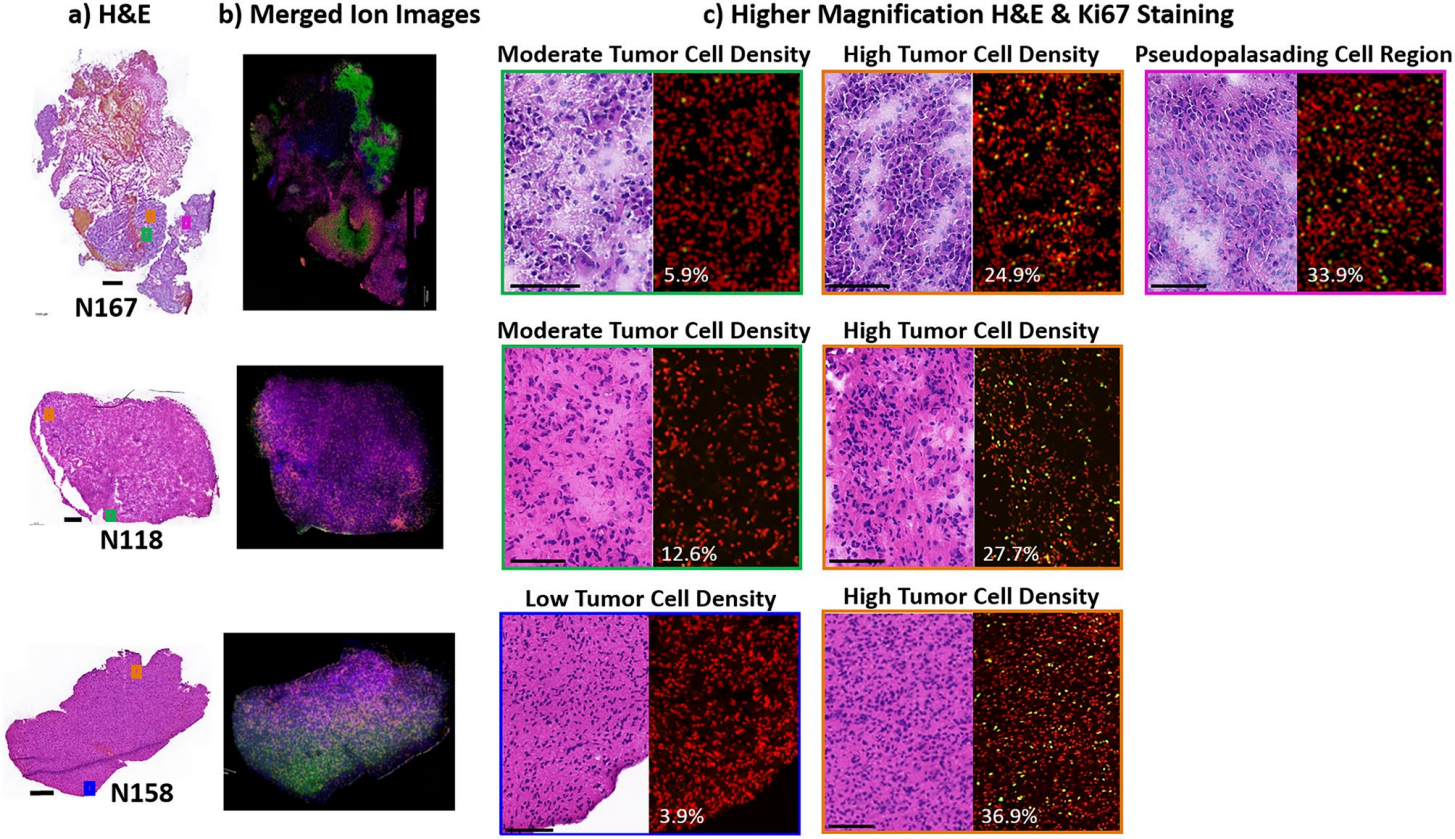
H&E-stained sections, merged ion images, and Ki67 staining with percent labeling index. (**a**) H&E-stained sections. Scale bars are 1 mm for N167 and 500 µm for samples N158 ans N118. (**b**) Merged ion images of GD3(36:1) at *m/*z 1470.830 in orange, PI(38:3) at *m/*z 887.562 is shown in pink, C1P(34:1) at *m/z* 616.471 in blue, and ST(42:2) at *m/z* 888.624 in green. (**c**) Higher magnification H&E images with corresponding Ki67 staining and percent labeling index. Hoechst nuclei (red); Ki67^+^ nuclei (green). Sections = 10 µm; scale bars are 100 µm.

Lipid ions that have the ability to discriminate between different tumor and endothelial cell regions in the glioblastoma TME have the potential to be biomarkers and as discussed in the previous sections, their pathways hold the potential to be targeted with therapeutics. To identify ions capable of discriminating between different tumor and endothelial cell subpopulations, additional studies were carried out to correlate histopathology and lipid distribution/accumulation to tumor cell proliferation, based on Ki67 labeling index (LI). As sample N141 contained mostly normal brain parenchyma and few tumor cell nuclei, this sample was excluded from further investigation. Serial sections were taken ~ 2–300 µm deeper from those discussed in the previous sections. MSI and Ki67 staining were carried out on adjacent sections. The deeper sections taken from patient sample N35 contained few tumor nuclei, more normal parenchyma, and both hemorrhage and necrosis due to disease and/or treatment. A few Ki67^+^ cells were detected in the top biopsy only and for these reasons, this sample was also excluded from further analysis. The stained H&E section and the adjacent Ki67 section for this sample is shown in Supplementary Fig. S7. The deeper sections taken from samples N167, N118 and N158 demonstrated comparable pathology and lipid profiles to those presented in the previous sections. Their H&E stained sections and representative MSI data are presented in Fig. 6a,b, respectively. The lipid ions presented for ST(42:2), C1P(34:1), GD3(36:1) and PI(38:3) in the merged ion images each demonstrate similar distributions and intensity to the previously analyzed sections (Fig. 5D). Higher magnification H&E images of low-moderate tumor cell density, high tumor cell density, and a region containing high tumor cell density with high numbers of pseudopalisading tumor cells are presented in Fig. 6c, along with the corresponding Ki67 staining and LI for each region. The Ki67 LI for each region indicates an increase in cell proliferation as tumor cell density increases. With the low-moderate tumor cell density regions containing < 13% LI and high tumor cell density regions containing > 25% LI. These findings correlate with those previously published evaluating Ki67 LI in patient glioblastoma samples^70^.

**Figure 7:**
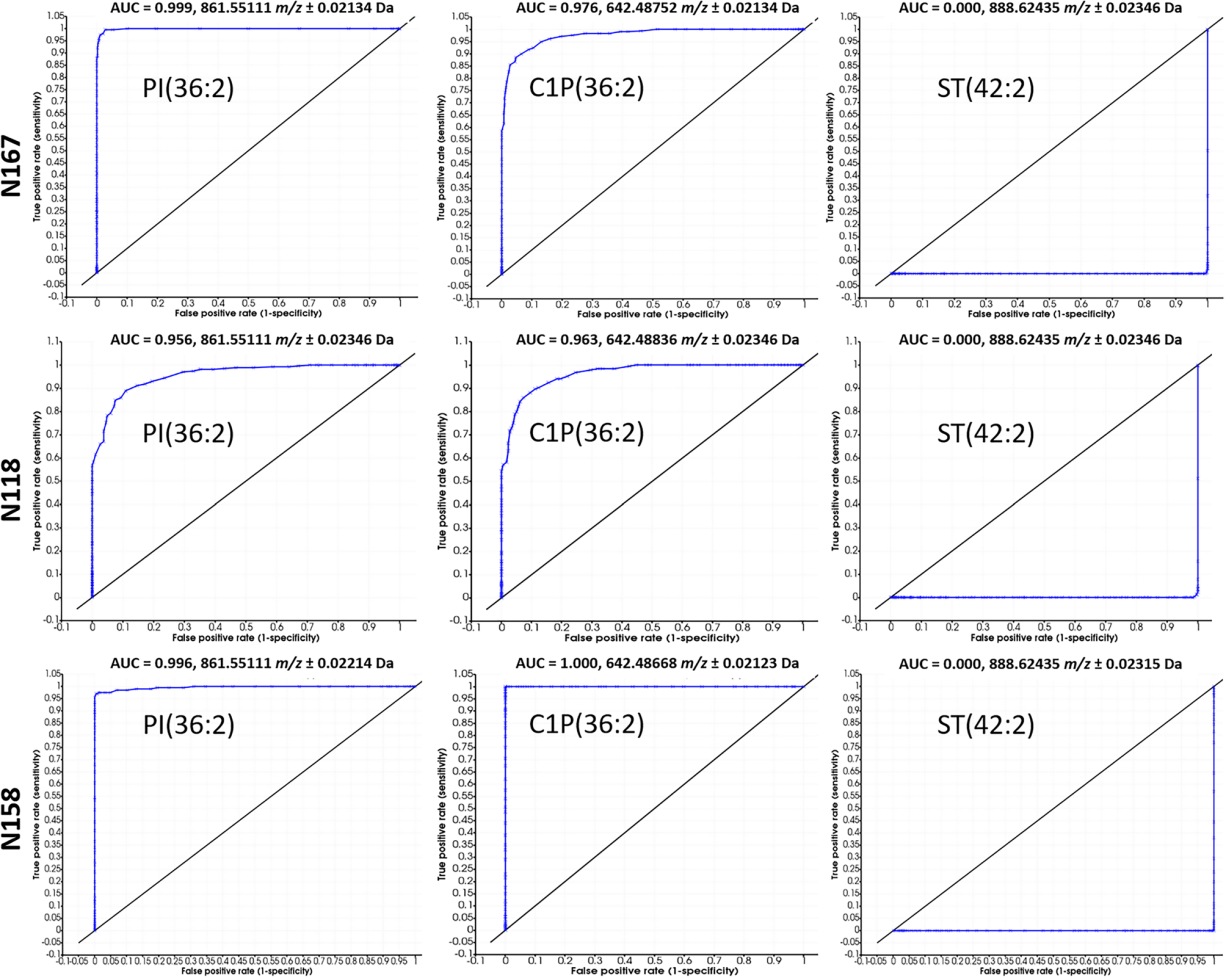
Example AUC—ROC plots for PI(36:2), C1P(36:2) and ST(42:2). Plots for N167 and N118 = high tumor cell density and high proliferation index vs. moderate tumor cell density and low proliferation index. Plots for N158 = high tumor cell density and high proliferation index vs. low tumor cell density and low proliferation index. PI(36:2) and C1P(36:2) (*m/z* 861.551 and 642.488 respectively) with AUC ≥ 0.956 discriminate high tumor cell density/high Ki67 regions, while ST(42:2) (*m/z* = 888.642) with AUC = 0 discriminates moderate and low tumor cell density regions/low Ki67 regions.

Regions of interest (ROIs) were identified in each paired patient sample based on PLSA data, histological profiles and in the latter sections, Ki67 LI. These encompassed areas with low-moderate tumor cell density and low cellular proliferation, high tumor cell density and proliferation, and the migratory pseudopalisading tumor cells. As the sample taken from N167 had large regions of high tumor cell density that were separated by the accumulation of the reported GSC marker, GD3, two high tumor cell regions were selected from this sample, covering GD3^High^ and GD3^Low^ areas. Ion that were able to discriminate high tumor cell density and palisading tumor cells from moderate-to-low tumor cell regions were calculated using area under the ROC curve, with a stringent cut off of 0.9. Only ions that met these criteria in all paired samples were accepted and their statistical significance was then calculated by *t *test. All discriminative ions had calculated *p-*values of < 0.001. The AUC values for each discriminative ion along with their calculated *p-*values are presented in Supplementary Table S13. While the number and type of discriminative ions differed in each sample, there were four lipids that could discriminate between all high tumor cell populations and the migratory pseudopalisading tumor cell regions, when compared to the low-moderate cellular areas. These were C1P(36:2), PI(36:2), PI(36:3) and PI(38:3). Example AUC-ROC graphs for several of these lipids along with one of the ST species that discriminated lower tumor cell regions are presented in Fig. 7. All ST species enabled discrimination of low-moderately-low tumor cell regions (data not shown). The similar fatty acyl chain compositions of lipids that discriminate all high tumor cell regions again indicate a potential structure–function relationship across all high tumor cell and migratory cell populations that warrants further investigation. The ability of cardiolipins to discriminate between high tumor cell regions but not the migratory pseudopalisading cellular regions is also of interest as this indicates differences in mitochondrial abundance and/or function between these different tumor cell subtypes. Zhang et al. demonstrated increased CL detection by MSI directly correlated with mitochondrial accumulation in oncocytic thyroid tumors when compared to non-oncocytic thyroid tumors^71^. Furthermore, mitochondrial numbers within cancer cells was recently shown to have a direct impact on drug response, in which increased numbers conferred cells more resistant to therapy. Understanding the differences in CL detection in relation to mitochondrial numbers and function, in the different glioblastoma cell populations, is thus an important next step of research as these cells may respond differently to therapy^72^.

A limitation of the current study was low sample number. Nevertheless, this is still an important pilot study as it is the first to map lipid profiles to regions of tumor cell subpopulations, including the migratory pseudopalisading tumor cells, in patient glioblastoma samples. It is also the first study to identify lipidomic differences within different formations of microvascular proliferations, which are known to aid in the aggressive nature of glioblastoma, and are being targeted with developmental chemotherapeutics.

In conclusion, high resolution MALDI MSI identified a number of lipids that differentiate tumor and endothelial cell subpopulations within human glioblastoma samples. The heterogeneous distribution of C1Ps, gangliosides, CLs and PIs within these cell populations further highlight the complexity of the glioblastoma TME. Lipid ions presented here lay the foundation for future studies that are required to understand their interconnecting signaling pathways in relation to cell function, tumor progression, and resistance to therapy. Understanding their functional relevance is essential for the identification of new therapeutics based on lipid pathway targets. In agreement with the known heterogeneity and clinical outcome of glioblastoma, data herein further suggests that targeting a single cell population with one treatment will not be efficacious in the battle to cure glioblastoma. The distribution of the pleotropic signaling lipid, C1P, within both tumor cell and MVP formations, along with their known roles in cell proliferation and recruitment, indicate that this lipid class may offer a novel duel therapeutic target for both tumor and endothelial cell populations. Whereas targeting GD3 with immunotherapeutics, or the mitochondrial-specific lipid, CLs, may only be efficacious in the treatment of glioblastoma tumor cell subclones. Targeting several of these lipids and their signaling pathways simultaneously, however, may improve clinical outcome. As targeting C1P, CL and gangliosides have shown efficacy in the treatment of many other cancers, their roles in glioblastoma progression is an important next step of research. Future work will investigate a larger cohort covering primary and recurrent tumors along with functional studies to understand the relevance of these findings.

## Methods

### Materials

Methanol, 9-aminoacridine (9AA), D.P.X, sodium chloride, donkey serum, gelatin from cold water fish skin, Trimza base for molecular biology, Tween 20 viscous liquid, Triton X-100 and bovine serum albumin were purchased from Sigma Aldrich (St. Louis, MO). Hematoxylin, eosin, xylene, ethanol, methanol-free formaldehyde, Alexa Fluor Plus 488 antibody and ultrapure glycerin were purchased from Thermo Fisher Scientific (Waltham, MA). Ki67 antibodies (ab15580) were purchased from Abcam (Cambridge, UK). TrueBlack autofluorescence quencher and Hoechst 33,342 dye were purchased from Biotium (Freemont, CA).

### Patient samples and ethics

Human glioblastoma samples were surgically resected at the Department of Neurosurgery, Hackensack Meridian Health (HMH), Hackensack, NJ, USA. Informed consent for the use of samples for scientific research was obtained from all patients according to the study protocol approved by the Institutional Review Board at Hackensack Meridian Health (Pro2018-1022). All procedures were performed in accordance with the Helsinki Declaration. Samples were de-identified and stored in HMH’s biorepository. All studies were conducted in accordance with the relevant guidelines and regulations, approved by the Ethics Review Committee of Hackensack Meridian Health.

### MSI sample preparation and data acquisition

Glioblastoma samples were sectioned to 5 or 10 µm thickness using a CM 1860 cryostat (Leica Microsystems; Buffalo Grove, IL), thaw mounted onto positively charged glass slides, and stored at – 80 °C until analysis. A 10 mg/mL solution of 9AA was sprayed onto the tissue samples using a TM Sprayer (HTX Technologies; Chapel Hill, NC). The spraying method was 160 µL/min at 80 °C for four passes using the crisscross setting, track spacing of 3 mm, nozzle height of 4 cm, and a velocity 1200 mm/min. Mass spectrometry imaging data were acquired using a 7 T SolariX 2xR FT-ICR mass spectrometer (Bruker Daltonics; Billerica, MA) operated in negative ion mode. Data were acquired with the small laser setting, accumulating 250 shots per pixel using the oversampling method^45^, resulting in an estimated pixel size of ~ 30 µm. Following data acquisition, the slides were washed and stained with H&E according to the manufacturer protocol (Thermo Fisher Scientific). Stained samples were annotated by a board-certified pathologist using CaseViewer software (version 2.4; 3D Histech).

### Ki67 staining

A 1 L solution of TBST was prepared by dissolving 24 g of Tris base and 88 g of sodium chloride in 1 L of water, adjusted to a pH of 7.6, and sterilized by autoclaving. Tissues were fixed with 4% formaldehyde and washed with TBST prior to staining. Sections were incubated for 1 h at room temperature in a blocking and permeabilization solution made up of 0.1% w/v gelatin, 2.25% w/v glycine and 1% w/v bovine serum albumin in 9:1 TBST:donkey serum. After 1 h, the blocking and permeabilization solution was removed and samples were incubated with 100 µL of Ki67 antibodies diluted 1:250 in 5% Triton X-100 in TBST. Samples were washed with TBST after removal of the antibody solution, and incubated in the secondary antibody solution (Alexa Fluor Plus 488 diluted 1:1000 in 5% Triton X-100 in TBST with Hoechst 33,342 counterstain added to a final concentration of 2 µg/mL). Finally, samples were incubated with an autofluorescence quenching solution for 1 min and washed with TBST again. Slides were mounted and left to dry overnight in the dark. Immunofluorescent imaging was carried out on a Nikon Eclipse Ti2 microscope using a 10 × objective. Ki67 positive cells were counted using the “Analyze Particles” function in ImageJ (version 1.8.0_172).

### Data analysis and statistical analysis

Data were analyzed using FlexImaging (version 5.0) and SCiLS Lab software (version 2021b MVS Pro; Bruker Daltonics). The H&E images and raw data for each patient sample were imported into the Scils lab software as individual files to investigate intratumor heterogeneity. Unsupervised multivariate analysis was carried out using probabilistic latent semantic analysis (PLSA) with deterministic initialization to identify regional trends in data^28^. PLSA data process was carried out without normalization, using individual spectra and 5, 7, 10 and 14 components were evaluated to determine the optimal number. The ions in each spectral loading were uploaded to LipidMaps and HMDB for tentative assignment. Regions of interest were defined based on the resulting score images that correlated to areas of low-moderate tumor cell density, different subpopulations of high tumor cell density, pseudopalisading tumor cells, and different formations of MVPs. Receiver operating characteristic (ROC) area under the curve (AUC) analysis was performed to find ions that discriminated between these regions. A stringent AUC cut off value of > 0.9 was used. The significance for each ion between two ROIs were calculated using the *t* test.

### Ethics approval and consent to participate

Tumor samples were collected by Hackensack Meridian Health’s Tissue Biorepository under the IRB protocol Pro2018-1022. Each participant gave written informed consent for the use of their tissue for scientific research and all work was carried out in compliance with the Helsinki Declaration.

## Supplementary Information

Supplementary Information.

## Data Availability

The datasets generated and/or analyzed during the current study will be made available from the corresponding author on reasonable request. Data will be uploaded to METASPACE (https://metaspace2020.eu/) upon publication.
