# Supplementary material for: Mass spectrometry imaging discriminates glioblastoma tumor cell subpopulations and different microvascular formations based on their lipid profiles
Source: Sci Rep. 2022 Oct 12;12:17069. doi: 10.1038/s41598-022-22093-4 (PMC9556690; doi:10.1038/s41598-022-22093-4)
Supplement: Supplementary file 1 — Supplementary Information. [file 41598_2022_22093_MOESM1_ESM.docx]

**Supplementary Information**

Mass spectrometry imaging discriminates glioblastoma tumor cell subpopulations and different microvascular formations based on their lipid profiles

Kelly C. O’Neill^1^, Evangelos Liapis^1^, Brent T. Harris^2^, David S. Perlin^1,3^, Claire L. Carter^1,4^

^1^Center for Discovery and Innovation, Hackensack Meridian Health, Nutley, New Jersey, 07110

^2^Departments of Neurology and Pathology, Georgetown University Medical Center, Washington D.C. 20007

^3^Department of Medical Sciences, Hackensack Meridian School of Medicine, Nutley, New Jersey, 07110

^4^Department of Pathology, Hackensack Meridian School of Medicine, Nutley, New Jersey, 07110

**Correspondence**

Claire L. Carter

Center for Discovery and Innovation

Hackensack Meridian Health

111 Ideation Way

Nutley, New Jersey, 07110

Phone: 201-880-3544

Email: [Claire.Carter@hmh-cdi.org](mailto:Claire.Carter@hmh-cdi.org)


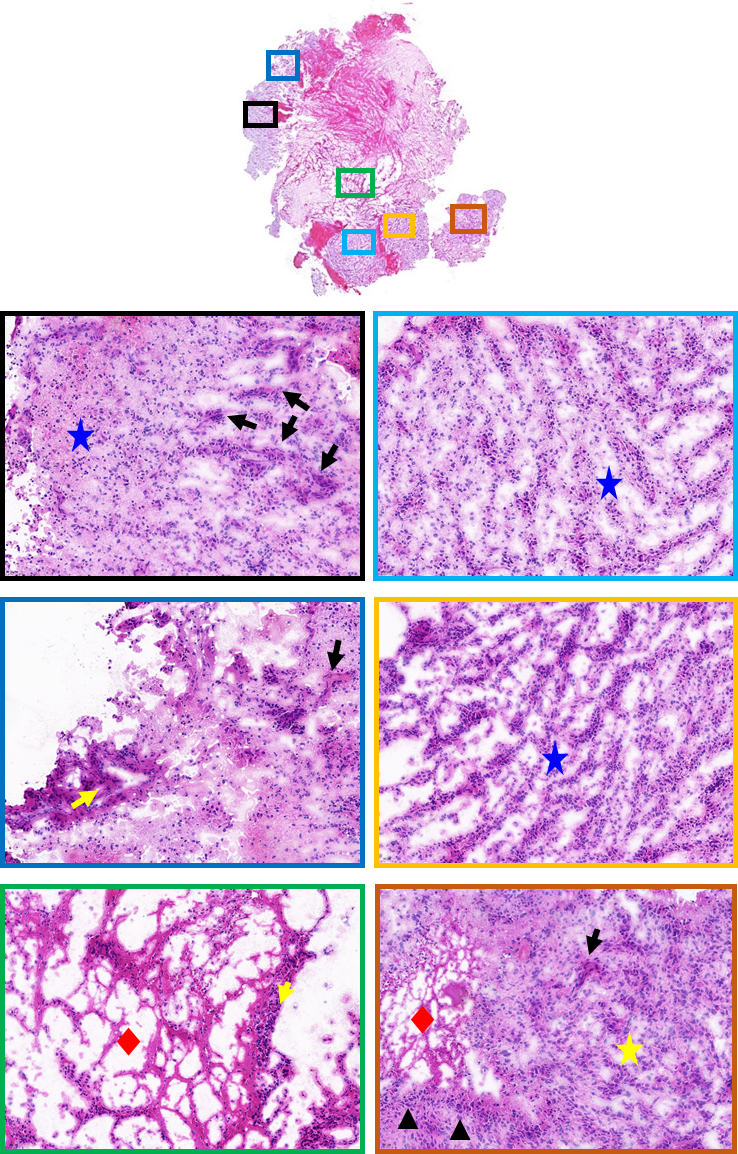


**Supplementary Figure S1**. H&E stained section taken from glioblastoma sample N167. High magnification images show regions of moderate tumor cell density (blue stars), high tumor cell density (yellow star) and palisading tumor cells (black arrow head) surrounding necrosis (red diamond). Microvascular proliferation with varying pathological presentations are shown (black arrows) and vascular garland microvascular formation (yellow arrows).


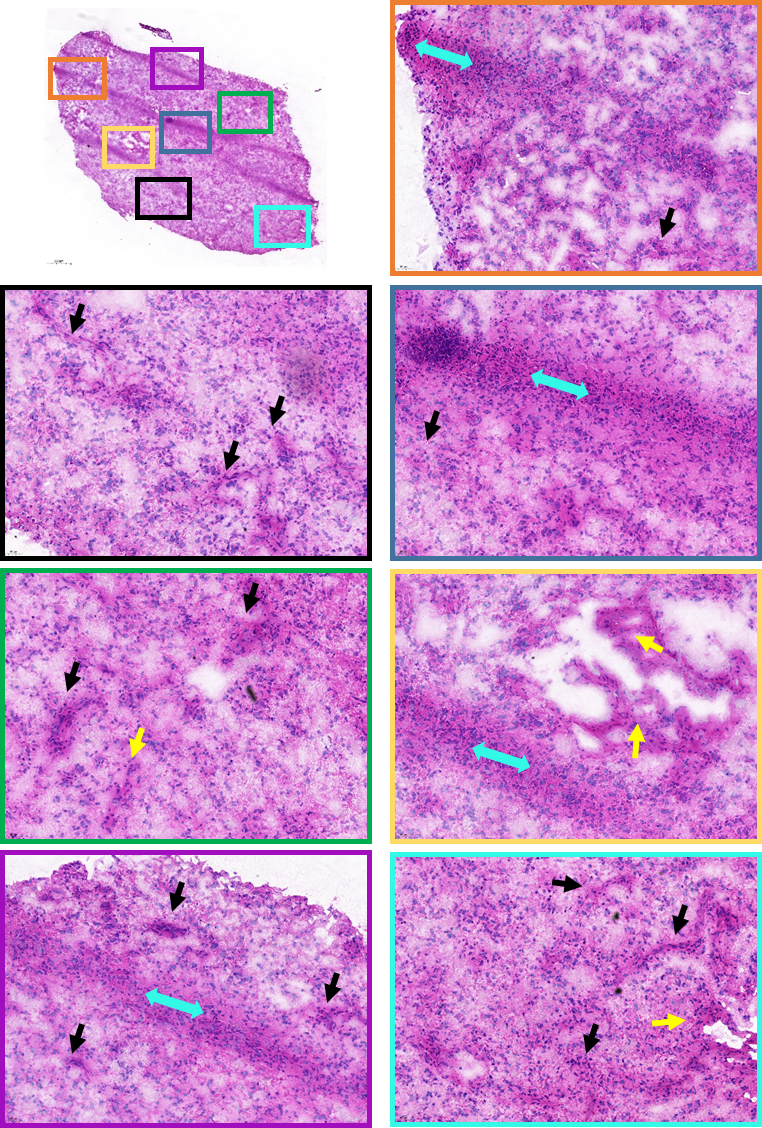


**Supplementary Figure S2**. H&E stained section taken from glioblastoma sample N118. High magnification regions demonstrate moderate tumor cell density and varying pathological presentations of microvascular formations throughout the section (black arrows). Vascular garland structures (yellow arrows) and bands of microvascular proliferation (turquoise doubles sided arrows) are shown.


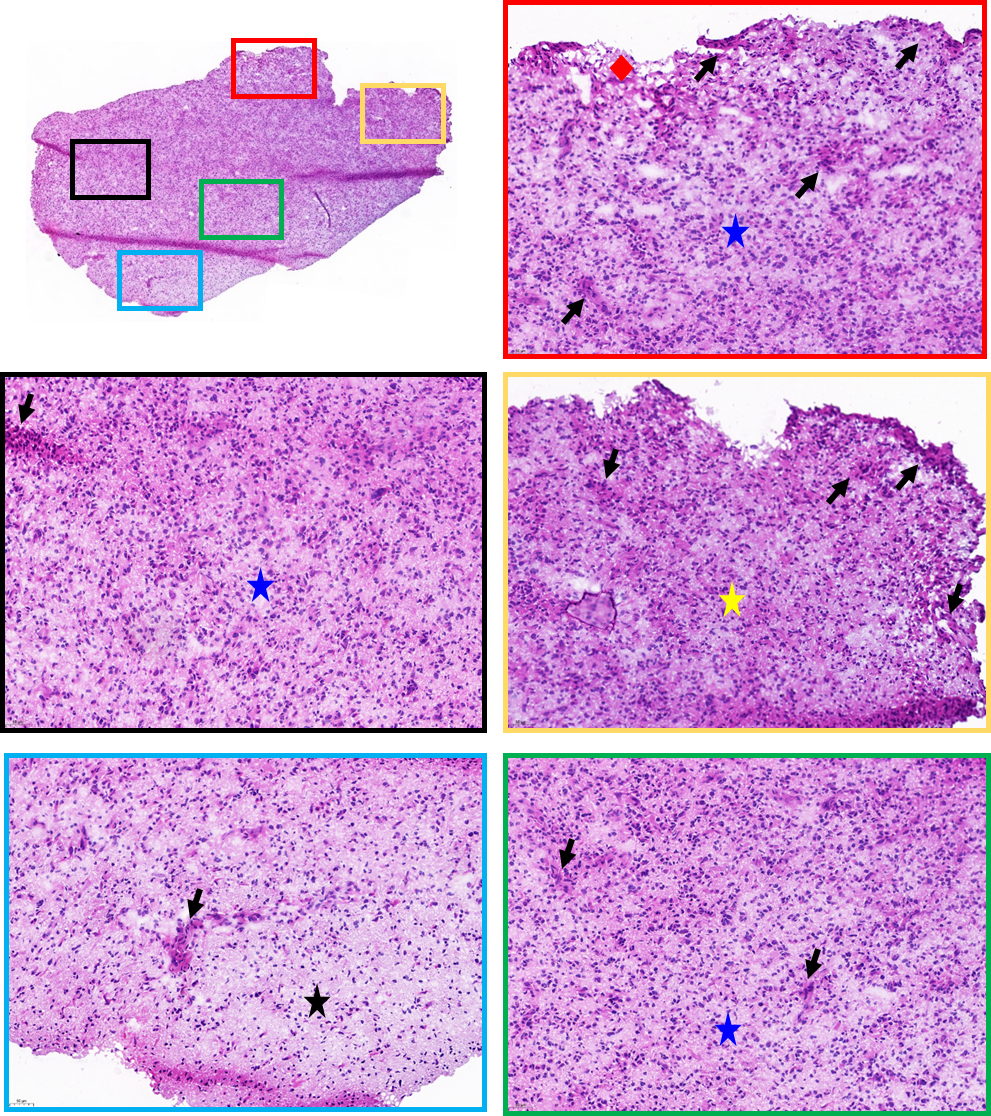


**Supplementary Figure S3**. H&E stained section taken from glioblastoma sample N158. High magnification regions demonstrate a gradient of low (black stars), to moderate (blue stars) and high (yellow star) regions of tumor cell density from the bottom to the top of the section. Regions of microvascular proliferation (black arrows) and necrosis (red diamond) are shown.


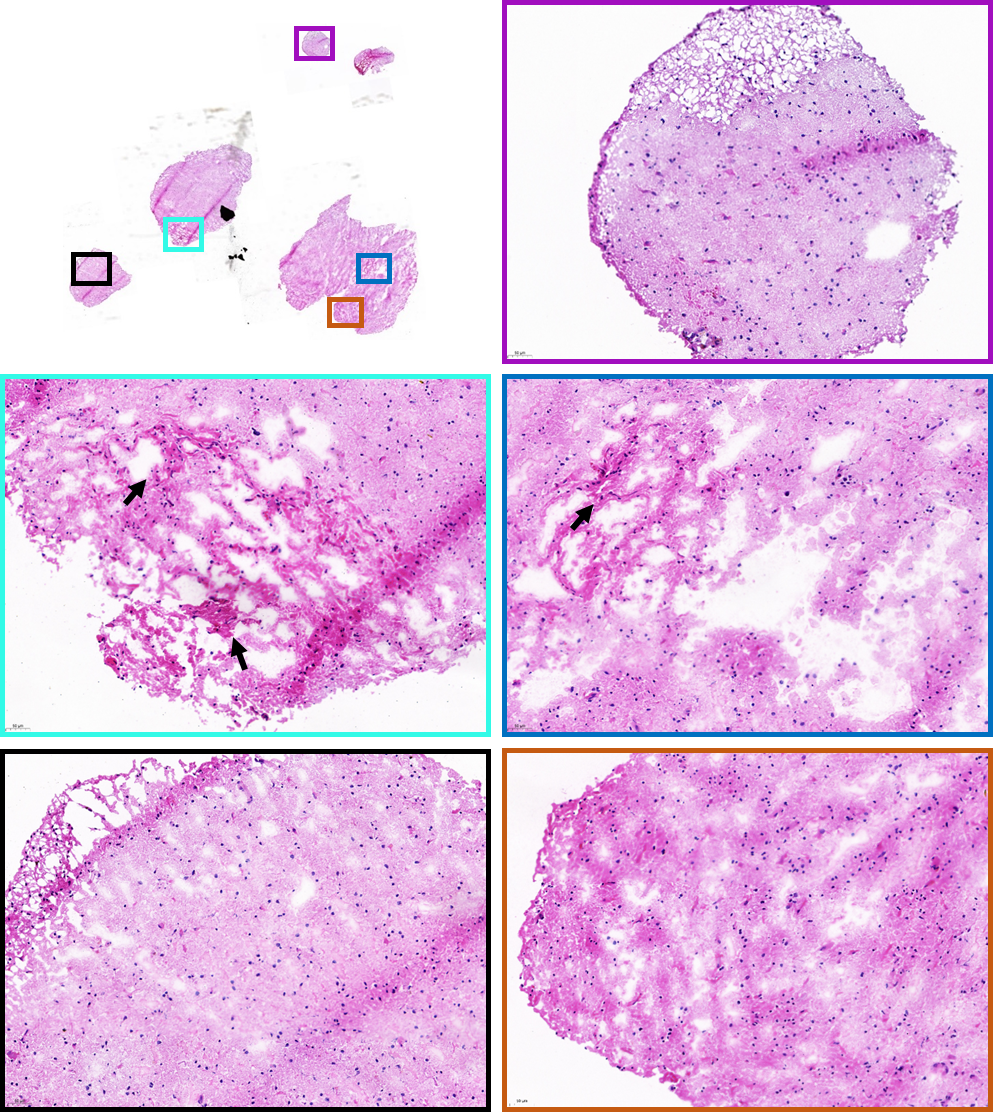


**Supplementary Figure S4**. H&E stained section taken from glioblastoma sample N141. High magnification regions demonstrate few tumor cells. Regions of microvascular proliferation (black arrows) are shown.


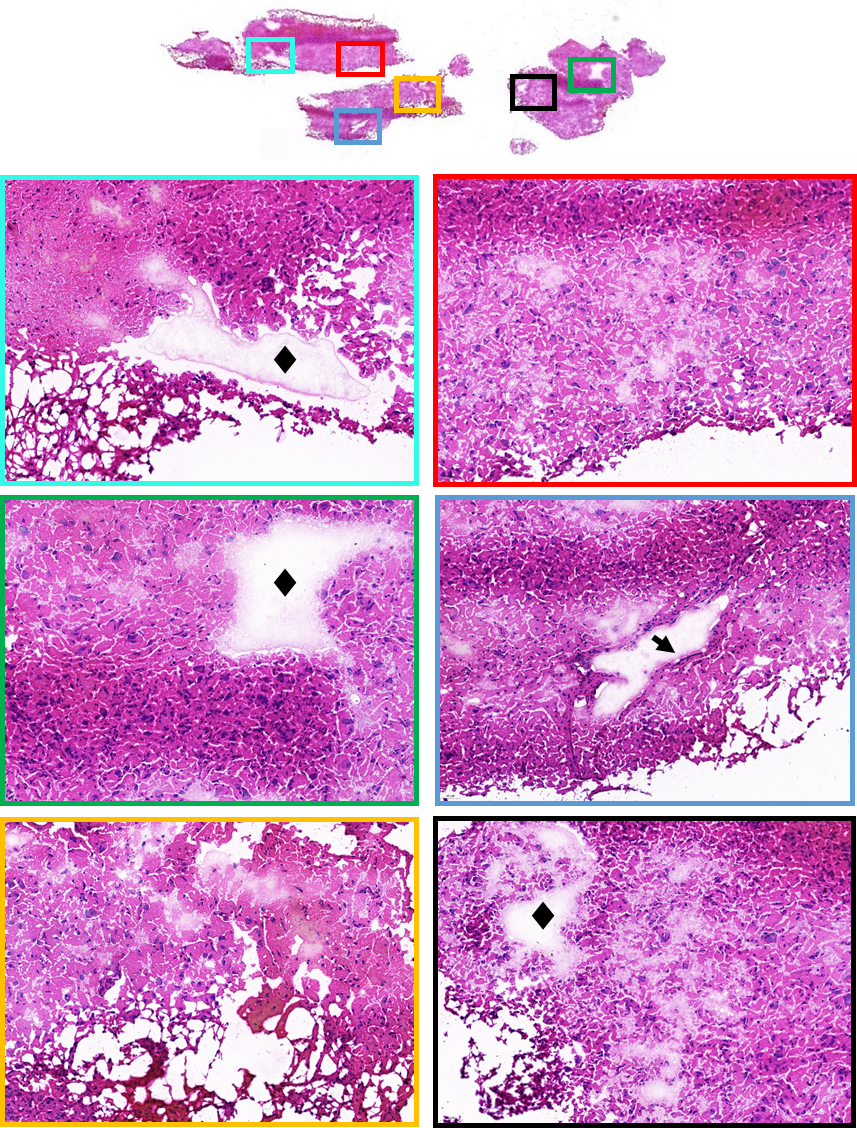


**Supplementary Figure S5**. H&E stained section taken from glioblastoma sample N35. High magnification regions demonstrate mild tumor infiltrate throughout the tissue. Areas of microvascular proliferation (black arrows) and acellular fluid-filled regions (black diamond) are shown.


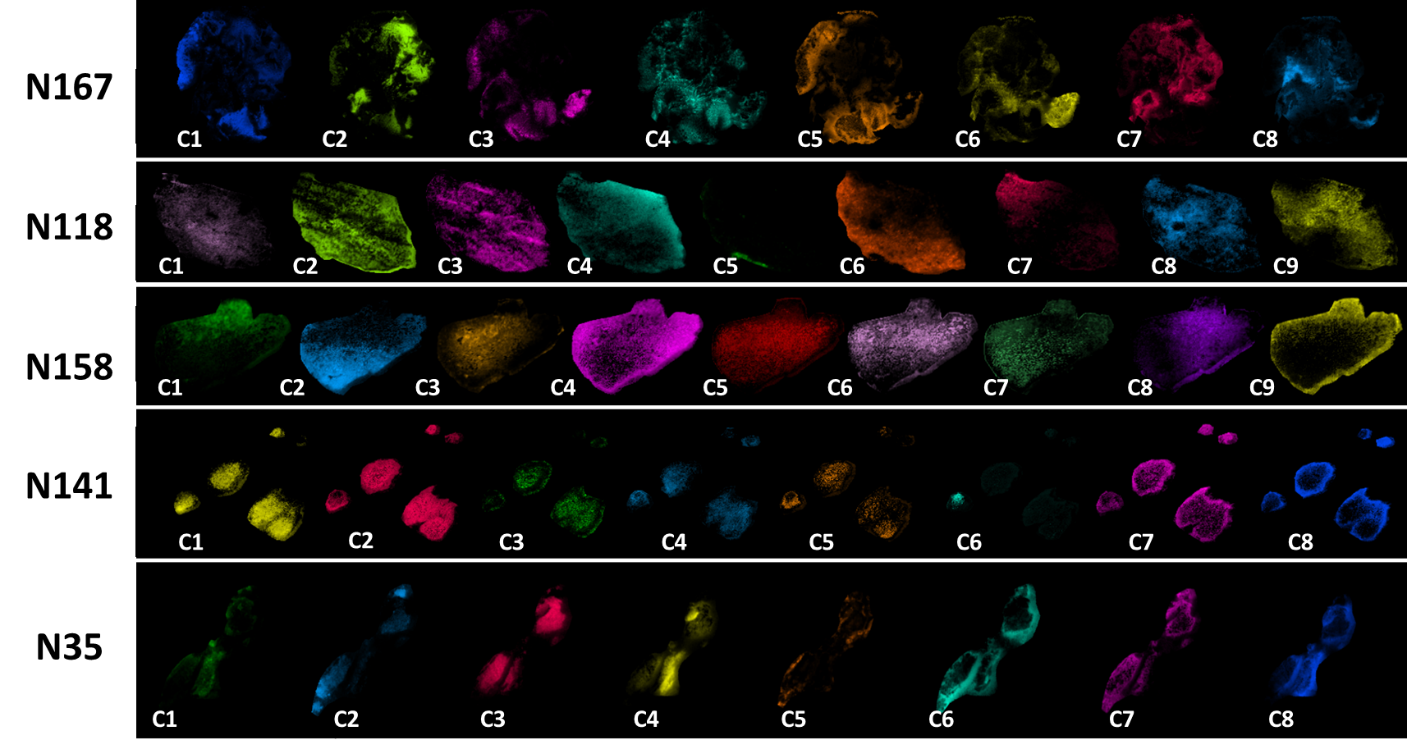


**Supplementary Figure S6**. PLSA score images display spatial features based on similarities and differences in mass spectra between tissues. The color schemes for each sample are separate for intra-tumor heterogeneity. Color intensity correlates to ion intensity for species in that component. The scale bars are 1 mm.


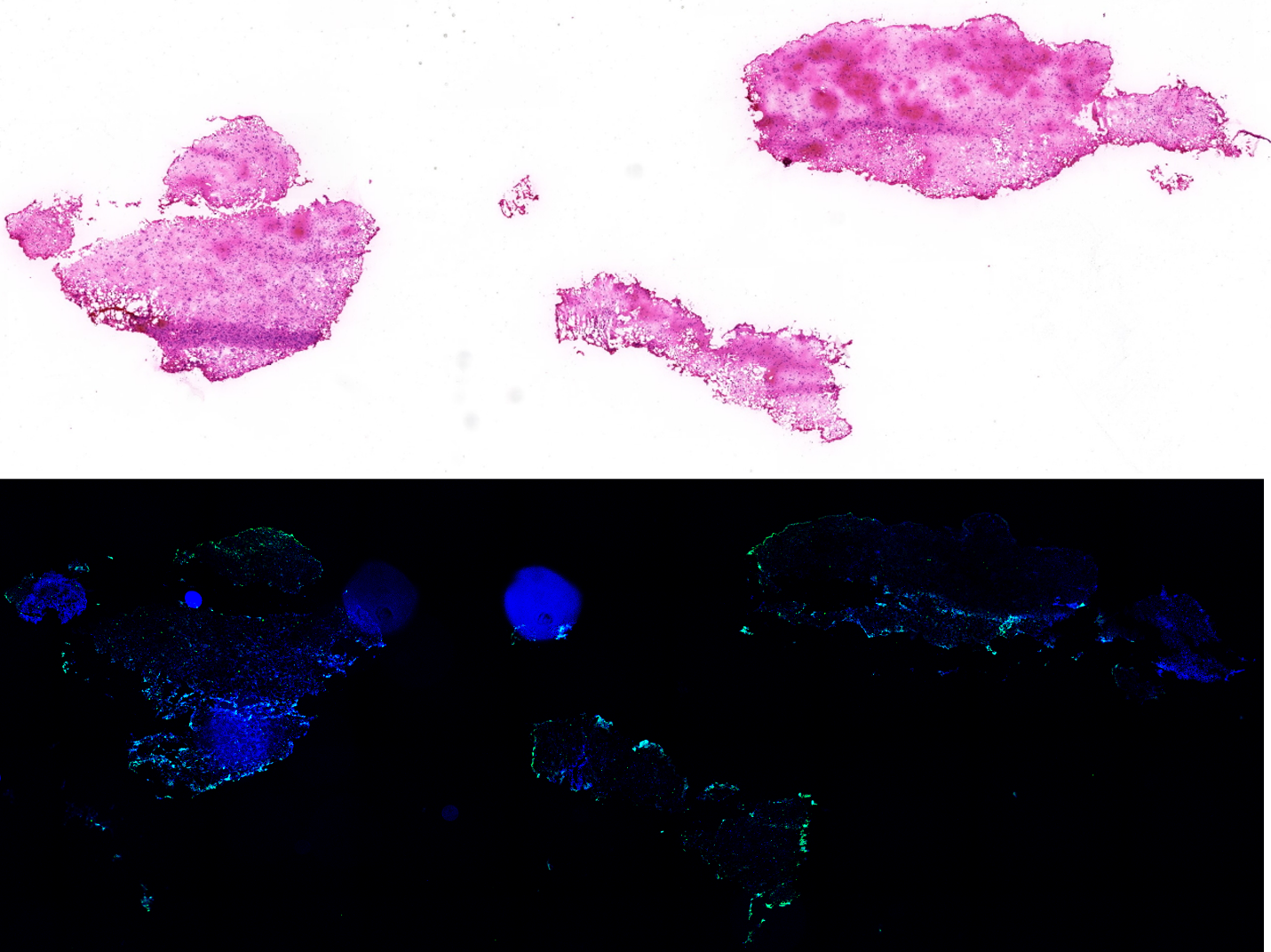
**Supplementary Figure S7**. H&E and Hoechst + Ki67 staining on the additional N35 section deeper in tissue block. Blue corresponds to Hoechst and green corresponds to Ki67 positive cells.

**Supplementary Table S1.** Tentatively identified cardiolipins and their distributions in different glioblastoma pathological regions. The number of asterisks corresponds to the signal intensity in that pathological region, with one asterisk for low signal intensity and three asterisks for high signal intensity.

| **Name** | **High Tumor Cell Density** | **Palisading Tumor Cells** | **Lower Tumor Cell Density** | **Vascular Garland** | **Sprouting MVP** | **Necrosis** | **Acellular Fluid** |
| --- | --- | --- | --- | --- | --- | --- | --- |
| CL (70:6) | N167***, N118**, N158**, N35** | N167*** |  |  | N118***, N158***, N35*** |  | N35** |
| CL (70:5) | N167***, N118**, N158***, N35*** | N167*** |  |  | N118***, N158***, N35*** |  | N35** |
| CL (70:4) | N167***, N118**, N158**, N35*** | N167*** |  |  | N118***, N158***, N35*** |  | N35** |
| CL (72:8) | N167***, N118**, N158***, N35*** | N167** |  | N167** | N167**, N118***, N158***, N35*** |  | N35* |
| CL (72:7) | N167***, N118**, N158***, N35*** | N167** |  | N167* | N167**, N118***, N158***, N35*** |  | N35** |
| CL (72:6) | N167***, N118***, N158***, N35*** | N167** |  |  | N167**, N118***, N158***, N35*** |  | N35** |
| CL (72:5) | N167***, N118***, N158***, N35*** | N167*** |  |  | N167**, N118***, N158***, N35*** |  | N35** |
| CL (74:10) | N167***, N118**, N158***, N35*** |  |  | N167* | N167***, N118***, N158***, N35*** |  | N35* |
| CL (74:9) | N167***, N118*, N158***, N35*** |  |  | N167* | N167***, N118***, N158***, N35*** |  | N35* |

**Supplementary Table S2.** Tentatively identified phosphatidylinositols and their distributions in different glioblastoma pathological regions. The number of asterisks corresponds to the signal intensity in that pathological region, with one asterisk for low signal intensity and three asterisks for high signal intensity.

| **Name** | **High Tumor Cell Density** | **Palisading Tumor Cells** | **Lower Tumor Cell Density** | **Vascular Garland** | **Sprouting MVP** | **Necrosis** | **Acellular Fluid** |
| --- | --- | --- | --- | --- | --- | --- | --- |
| [PI (32:0](https://www.lipidmaps.org/data/Gsn_expand.php?ABBREV=PI(32:0)&even=2)) | N167*, N118**, N158***, N35*** |  | N158**, N141**, N35* |  | N167***, N118***, N158***, N141***, N35*** |  | N35*** |
| [PI (34:2](https://www.lipidmaps.org/data/Gsn_expand.php?ABBREV=PI(34:2)&even=2)) | N167***, N118**, N158***, N35*** | N167** | N158**, N141***, N35* |  | N167***, N118***, N158***, N141***, N35*** |  | N35** |
| [PI (34:1](https://www.lipidmaps.org/data/Gsn_expand.php?ABBREV=PI(34:1)&even=2)) | N167***, N118***, N158***, N35*** | N167*** | N158**, N141*** |  | N167***, N118***, N158***, N141***, N35*** |  | N35** |
| [PI (34:0](https://www.lipidmaps.org/data/Gsn_expand.php?ABBREV=PI(34:0)&even=2)) | N167*, N118***, N158***, N35*** | N167* | N158**, N141*** |  | N167***, N118***, N158***, N141***, N35*** |  | N35** |
| [PI (36:5](https://www.lipidmaps.org/data/Gsn_expand.php?ABBREV=PI(36:5)&even=2)) | N167***, N118***, N158**, N35** |  | N158** |  | N118***, N35*** |  | N35** |
| [PI (36:4](https://www.lipidmaps.org/data/Gsn_expand.php?ABBREV=PI(36:4)&even=2)) | N167***, N118**, N158***, N35*** |  | N158**, N141*** | N167* | N118***, N158***, N141**, N35*** |  | N35** |
| [PI (36:3](https://www.lipidmaps.org/data/Gsn_expand.php?ABBREV=PI(36:3)&even=2)) | N167***, N118**, N158***, N35*** | N167*** | N158**, N141*** |  | N118***, N158***, N141**, N35*** |  | N35** |
| [PI (36:2](https://www.lipidmaps.org/data/Gsn_expand.php?ABBREV=PI(36:2)&even=2)) | N167***, N118**, N158***, N35*** | N167** | N158**, N141***, N35* |  | N167***, N118***, N158***, N141**, N35*** |  | N35** |
| [PI (36:1](https://www.lipidmaps.org/data/Gsn_expand.php?ABBREV=PI(36:1)&even=2)) | N167**, N118**, N158***, N35*** | N167** | N158**, N141*** |  | N167***, N118***, N158***, N141**, N35** |  | N35** |
| [PI (38:6](https://www.lipidmaps.org/data/Gsn_expand.php?ABBREV=PI(38:6)&even=2)) | N167***, N118**, N158***, N35*** | N167*** | N141* |  | N167**, N118***, N158***, N141*, N35*** |  | N35** |
| [PI (38:5](https://www.lipidmaps.org/data/Gsn_expand.php?ABBREV=PI(38:5)&even=2)) | N167***, N118**, N158***, N35*** |  | N158**, N141*** | N167* | N167**, N118***, N158***, N141***, N35*** |  | N35** |
| [PI (38:4](https://www.lipidmaps.org/data/Gsn_expand.php?ABBREV=PI(38:4)&even=2)) | N167***, N118**, N158***, N35*** | N167** | N158**, N141*** |  | N167**, N118***, N158***, N141***, N35*** |  | N35*** |
| [PI (38:3](https://www.lipidmaps.org/data/Gsn_expand.php?ABBREV=PI(38:3)&even=2)) | N167**, N118**, N158***, N35*** | N167*** | N141*** |  | N167**, N118***, N158***, N141***, N35*** |  | N35** |
| [PI (40:6](https://www.lipidmaps.org/data/Gsn_expand.php?ABBREV=PI(40:6)&even=2)) | N167***, N118*, N158***, N35*** | N167*** | N141*** |  | N167***, N118***, N158***, N141***, N35*** |  | N35** |
| [PI (40:5](https://www.lipidmaps.org/data/Gsn_expand.php?ABBREV=PI(40:5)&even=2)) | N167**, N118*, N158***, N35*** | N167*** | N141* |  | N167***, N118***, N158***, N35*** |  | N35** |
| [PI (40:4](https://www.lipidmaps.org/data/Gsn_expand.php?ABBREV=PI(40:4)&even=2)) | N167**, N118*, N158***, N35*** | N167* | N141*** |  | N167***, N118***, N158***, N141**, N35*** |  | N35** |

**Supplementary Table S3.** Tentatively identified sphingomyelins and their distributions in different glioblastoma pathological regions. The number of asterisks corresponds to the signal intensity in that pathological region, with one asterisk for low signal intensity and three asterisks for high signal intensity.

| **Name** | **High Tumor Cell Density** | **Palisading Tumor Cells** | **Lower Tumor Cell Density** | **Vascular Garland** | **Sprouting MVP** | **Necrosis** | **Acellular Fluid** |
| --- | --- | --- | --- | --- | --- | --- | --- |
| SM (34:2) | N167**, N118*, N158* |  | N158***, N141** |  | N118*, N141** | N167*** |  |
| SM (34:1) | N118*, N158**, N35* |  | N141**, N35*** | N118*** | N118***, N158**, N141***, N35*** | N167***, N158**, N35*** | N35*** |
| SM (36:2) | N118**, N35** |  | N35** | N118* | N118***, N158**, N141**, N35*** | N167***, N158** | N35*** |
| SM (36:1) | N167*, N118*, N157**, N35* |  | N141*, N35* | N167* | N167**, N118***, N158**, N141***, N35*** | N167***, N158**, N35*** | N35*** |
| SM (38:1) |  |  |  | N118*** | N167**, N35*** | N167***, N35** | N35*** |
| SM (40:2) |  |  |  | N118*** | N167** | N167*** |  |
| SM (40:1) |  |  |  | N167*, N118*** | N167**, N118* | N167*** |  |
| SM (42:3) |  |  |  | N118*** | N167*** | N167*** |  |
| SM (42:2) | N118**, N158**, N35* |  | N158***, N141**, N35*** | N118*** | N167***, N118**, N158**, N141***, N35** | N167***, N158**, N35** | N35*** |
| SM (42:1) |  |  |  | N118*** | N167***, N118*, N141** | N167*** |  |

**Supplementary Table S4.** Tentatively identified ceramide-1-phosphates and their distributions in different glioblastoma pathological regions. The number of asterisks corresponds to the signal intensity in that pathological region, with one asterisk for low signal intensity and three asterisks for high signal intensity.

| **Name** | **High Tumor Cell Density** | **Palisading Tumor Cells** | **Lower Tumor Cell Density** | **Vascular Garland** | **Sprouting MVP** | **Necrosis** | **Acellular Fluid** |
| --- | --- | --- | --- | --- | --- | --- | --- |
| C1P (34:1) | N118***, N158***, N35** |  | N168**, N141**, N35** | N118*** | N167***, N118***, N158***, N141***, N35*** | N167***, N158**, N35** | N35*** |
| C1P (36:2) | N167*, N118***, N158***, N35** | N167* | N158***, N141**, N35** | N118*** | N167***, N118***, N158***, N141***, N35*** | N167***, N158***, N35** | N35*** |
| C1P (36:1) | N167**, N118***, N158***, N35** | N167*** | N158**, N141***, N35* | N167*, N118** | N167***, N118***, N158***, N141***, N35*** | N167***, N158**, N35* | N35*** |
| C1P (38:3) | N118***, N158**, N35** | N167* | N158***, N141** | N118** | N167***, N118***, N158***, N141***, N35*** | N167***, N158*** | N35*** |
| C1P (38:2) | N167**, N118***, N158**, N35** | N167*** | N158***, N141*** | N167*, N118* | N167***, N118***, N158***, N141***, N35*** | N167***, N158***, N35** | N35*** |
| C1P (42:2) | N118**, N35* |  | N158***, N141***, N35*** | N118*** | N167***, N118**, N141*** | N167*** |  |
| C1P (42:1) | N118*, N35** |  | N158***, N141***, N35*** | N118*** | N167***, N118*, N141***, N35* | N167***, N158*** | N35** |
| C1P (44:3) | N118**, N35** | N167** | N158***, N141***, N35*** | N167*, N118*** | N167***, N118***, N141**, N35*** | N167***, N158***, N35** | N35*** |

**Supplementary Table S5.** Tentatively identified gangliosides and their distributions in different glioblastoma pathological regions. The number of asterisks corresponds to the signal intensity in that pathological region, with one asterisk for low signal intensity and three asterisks for high signal intensity.

| **Name** | **High Tumor Cell Density** | **Palisading Tumor Cells** | **Lower Tumor Cell Density** | **Vascular Garland** | **Sprouting MVP** | **Necrosis** | **Acellular Fluid** |
| --- | --- | --- | --- | --- | --- | --- | --- |
| GM3 (36:1) | N167**, N118***, N158**, N35*** |  |  |  | N167**, N118***, N158***, N35*** | N167*** | N35** |
| GM3 (40:1) | N167**, N118*** |  |  |  | N167***, N118***, N158* | N167*** |  |
| GM3 (42:2) | N167*, N118***, N158** |  | N141*** |  | N167***, N118***, N158*** | N167*** |  |
| GM2 (34:1) | N167**, N158*** |  | N158* |  | N167**, N158*** | N167*** |  |
| GM2 (36:1) | N167**, N118***, N35** |  |  |  | N167***, N118**, N35*** | N167*** | N35*** |
| GM2 (38:1) | N167***, N118***, N158**, N35** | N167** |  |  | N167**, N118***, N158***, N35** | N167*** | N35** |
| GM2 (42:1) | N167*, N118**, N158** |  |  |  | N167***, N158*** | N167*** |  |
| GD3 (36:1) | N167***, N118***, N158***, N35** | N167** |  |  | N167**, N118**, N158***, N35*** | N167*** | N35*** |
| GM1 (36:1) | N167*, N118***, N158**, N35*** | N167** | N158*, N141** |  | N167***, N118**, N158***, N35** | N167*** | N35*** |
| GM1 (38:1) | N167*, N158**, N35* |  | N158*, N141*** |  | N158***, N35* | N167*** | N35*** |

**Supplementary Table S6.** Tentatively identified sulfatides and their distributions in different glioblastoma pathological regions. The number of asterisks corresponds to the signal intensity in that pathological region, with one asterisk for low signal intensity and three asterisks for high signal intensity.

| **Name** | **High Tumor Cell Density** | **Palisading Tumor Cells** | **Lower Tumor Cell Density** | **Vascular Garland** | **Sprouting MVP** | **Necrosis** | **Acellular Fluid** |
| --- | --- | --- | --- | --- | --- | --- | --- |
| ST (36:1) | N35* |  | N167***, N118***, N158***, N141***, N35*** |  | N118***, N158***, N141** | N167*** |  |
| ST (38:1) | N158**, N35** |  | N167***, N158***, N141***, N35* | N167*** | N167***, N158***, N141***, N35*** | N167*** | N35** |
| ST (40:2) | N35* |  | N167**, N118***, N158***, N141***, N35*** | N167*** | N167***, N118***, N158***, N35* | N167*** | N35* |
| ST (40:1) | N167**, N35** |  | N167***, N118***, N158***, N141***, N35*** | N167** | N167**, N118***, N158***, N35* | N167*** | N35* |
| ST (40:0) | N167**, N158** |  | N167***, N158***, N141*** | N167*** | N167***, N158***, N35** | N167*** | N35** |
| ST (42:4) | N167***, N118**, N158***, N35*** |  | N167***, N158**, N141**, N35* |  | N167***, N118***, N158**, N141**, N35*** | N167** | N35** |
| ST (42:3) | N167**, N158**, N35** |  | N167***, N158***, N141***, N35*** |  | N167**, N158**, N35** | N167*** | N35* |
| ST (42:2) | N167**, N35** |  | N167**, N118***, N158***, N141***, N35*** |  | N167**, N158***, N35* | N167*** | N36* |
| ST (42:1) | N167**, N35** |  | N167***, N118***, N158***, N141***, N35*** | N167** | N167**, N158*** | N167*** | N35* |
| ST (42:0) | N167**, N35*** |  | N167**, N118***, N158***, N141***, N35*** | N167** | N167**, N158***, N35** | N167*** | N35** |
| ST (44:5) | N167**, N118***, N158***, N35*** | N167*** | N118***, N158***, N141***, N35* |  | N167**, N118***, N158***, N35*** | N167*** | N35** |
| ST (44:4) | N118**, N158**, N35** |  | N158**, N141**, N35* |  | N167***, N118***, N158***, N141**, N35*** | N167*** | N35** |
| ST (44:3) | N167**, N118*, N35** |  | N167**, N118***, N158***, N141***, N35*** | N167** | N167***, N118***, N158***, N35** | N167*** | N35* |
| ST (44:2) | N167**, N35** |  | N167**, N118***, N158***, N141***, N35*** |  | N167**, N158***, N35* | N167*** | N35* |
| ST (44:1) | N167**, N35*** |  | N167**, N118***, N158***, N141***, N35*** |  | N167**, N158**, N35*** | N167*** | N35** |
| ST (46:6) | N167**, N118***, N35*** |  | N167**, N118**, N158***, N141***, N35** | N167**, N118** | N167**, N118***, N158***, N141***, N35** | N167***, N158***, N35* | N35** |
| ST (46:5) | N167*, N118**, N35*** |  | N167*, N118**, N158**, N141***, N35** |  | N167*, N118***, N158***, N141***, N35*** | N167***, N158**, N35* | N35** |
| ST (48:6) | N167*, N118**, N35** |  | N167*, N118**, N141*, N35** |  | N167**, N118***, N141* | N167*** |  |
| ST (48:5) | N118*, N35** |  | N167*, N141*, N35** |  | N167**, N118***, N141*, N35** | N167*** | N35** |

**Supplementary Table S7**. Tentatively identified cardiolipins.

| **Exp. Mass** | **Exact Mass** | **Error (ppm)** | **Name** | **Formula** | **Adduct** |
| --- | --- | --- | --- | --- | --- |
| 1423.9656 | 1423.9650 | 0.39 | CL (70:6) | C_79_H_142_O_17_P_2_ | [M-H]- |
| 1425.9839 | 1425.9806 | 2.34 | CL (70:5) | C_79_H_144_O_17_P_2_ | [M-H]- |
| 1427.9972 | 1427.9963 | 0.61 | CL (70:4) | C_79_H_146_O_17_P_2_ | [M-H]- |
| 1449.9781 | 1449.9806 | 1.73 | CL (72:7) | C_81_H_144_O_17_P_2_ | [M-H]- |
| 1451.9951 | 1451.9963 | 0.85 | CL (72:6) | C_81_H_146_O_17_P_2_ | [M-H]- |
| 1454.0108 | 1454.0119 | 0.76 | CL (72:5) | C_81_H_148_O_17_P_2_ | [M-H]- |
| 1471.9656 | 1471.9650 | 0.39 | CL (74:10) | C_83_H_142_O_17_P_2_ | [M-H]- |
| 1473.9809 | 1473.9806 | 0.22 | CL (74:9) | C_83_H_144_O_17_P_2_ | [M-H]- |

**Supplementary Table S8**. Tentatively identified phosphatidylinositols.

| **Exp. Mass** | **Exact Mass** | **Error (ppm)** | **Name** | **Formula** | **Adduct** |
| --- | --- | --- | --- | --- | --- |
| 809.5204 | 809.5186 | 2.35 | [PI (32:0](https://www.lipidmaps.org/data/Gsn_expand.php?ABBREV=PI(32:0)&even=2)) | C_41_H_79_O_13_P | [M-H]- |
| 833.5210 | 833.5186 | 2.28 | [PI (34:2](https://www.lipidmaps.org/data/Gsn_expand.php?ABBREV=PI(34:2)&even=2)) | C_43_H_79_O_13_P | [M-H]- |
| 835.5365 | 835.5342 | 2.27 | [PI (34:1](https://www.lipidmaps.org/data/Gsn_expand.php?ABBREV=PI(34:1)&even=2)) | C_43_H_81_O_13_P | [M-H]- |
| 837.5519 | 837.5499 | 2.27 | [PI (34:0](https://www.lipidmaps.org/data/Gsn_expand.php?ABBREV=PI(34:0)&even=2)) | C_43_H_83_O_13_P | [M-H]- |
| 855.5038 | 855.5029 | 2.22 | [PI (36:5](https://www.lipidmaps.org/data/Gsn_expand.php?ABBREV=PI(36:5)&even=2)) | C_45_H_77_O_13_P | [M-H]- |
| 857.5197 | 857.5186 | 2.22 | [PI (36:4](https://www.lipidmaps.org/data/Gsn_expand.php?ABBREV=PI(36:4)&even=2)) | C_45_H_79_O_13_P | [M-H]- |
| 859.5354 | 859.5342 | 2.21 | [PI (36:3](https://www.lipidmaps.org/data/Gsn_expand.php?ABBREV=PI(36:3)&even=2)) | C_45_H_81_O_13_P | [M-H]- |
| 861.5511 | 861.5499 | 2.22 | [PI (36:2](https://www.lipidmaps.org/data/Gsn_expand.php?ABBREV=PI(36:2)&even=2)) | C_45_H_83_O_13_P | [M-H]- |
| 863.5642 | 863.5655 | 2.20 | [PI (36:1](https://www.lipidmaps.org/data/Gsn_expand.php?ABBREV=PI(36:1)&even=2)) | C_45_H_85_O_13_P | [M-H]- |
| 881.5204 | 881.5186 | 2.16 | [PI (38:6](https://www.lipidmaps.org/data/Gsn_expand.php?ABBREV=PI(38:6)&even=2)) | C_47_H_79_O_13_P | [M-H]- |
| 883.5345 | 883.5342 | 2.15 | [PI (38:5](https://www.lipidmaps.org/data/Gsn_expand.php?ABBREV=PI(38:5)&even=2)) | C_47_H_81_O_13_P | [M-H]- |
| 885.5502 | 885.5499 | 2.15 | [PI (38:4](https://www.lipidmaps.org/data/Gsn_expand.php?ABBREV=PI(38:4)&even=2)) | C_47_H_83_O_13_P | [M-H]- |
| 887.5624 | 887.5655 | 2.14 | [PI (38:3](https://www.lipidmaps.org/data/Gsn_expand.php?ABBREV=PI(38:3)&even=2)) | C_47_H_85_O_13_P | [M-H]- |
| 909.5517 | 909.5499 | 2.09 | [PI (40:6](https://www.lipidmaps.org/data/Gsn_expand.php?ABBREV=PI(40:6)&even=2)) | C_49_H_83_O_13_P | [M-H]- |
| 911.5697 | 911.5655 | 2.08 | [PI (40:5](https://www.lipidmaps.org/data/Gsn_expand.php?ABBREV=PI(40:5)&even=2)) | C_49_H_85_O_13_P | [M-H]- |
| 913.5859 | 913.5812 | 2.08 | [PI (40:4](https://www.lipidmaps.org/data/Gsn_expand.php?ABBREV=PI(40:4)&even=2)) | C_49_H_87_O_13_P | [M-H]- |

**Supplementary Table S9**. Tentatively identified ceramide-1-phosphates.

| **Exp. Mass** | **Exact Mass** | **Error (ppm)** | **Name** | **Formula** | **Adduct** |
| --- | --- | --- | --- | --- | --- |
| 616.4722 | 616.4712 | 1.78 | C1P (34:1) | C_34_H_68_NO_6_P | [M-H]- |
| 642.4875 | 642.4868 | 1.71 | C1P (36:2) | C_36_H_70_NO_6_P | [M-H]- |
| 644.5018 | 644.5025 | 1.71 | C1P (36:1) | C_36_H_72_NO_6_P | [M-H]- |
| 668.5027 | 668.5025 | 1.65 | C1P (38:3) | C_38_H_72_NO_6_P | [M-H]- |
| 670.5185 | 670.5181 | 1.64 | C1P (38:2) | C_38_H_74_NO_6_P | [M-H]- |
| 726.5813 | 726.5807 | 1.51 | C1P (42:2) | C_42_H_82_NO_6_P | [M-H]- |
| 728.5980 | 728.5964 | 1.51 | C1P (42:1) | C_42_H_84_NO_6_P | [M-H]- |
| 752.5975 | 752.5964 | 1.46 | C1P (44:3) | C_44_H_84_NO_6_P | [M-H]- |

**Supplementary Table S10**. Tentatively identified sphingomyelins.

| **Exp. Mass** | **Exact Mass** | **Error (ppm)** | **Name** | **Formula** | **Adduct** |
| --- | --- | --- | --- | --- | --- |
| 685.5294 | 685.529 | 1.60 | SM (34:2) | C_39_H_77_N_2_O_6_P | [M-CH3]- |
| 687.5461 | 687.5447 | 1.60 | SM (34:1) | C_39_H_79_N_2_O_6_P | [M-CH3]- |
| 713.5616 | 713.5603 | 1.54 | SM (36:2) | C_41_H_81_N_2_O_6_P | [M-CH3]- |
| 715.5778 | 715.576 | 1.54 | SM (36:1) | C_41_H_83_N_2_O_6_P | [M-CH3]- |
| 743.6081 | 743.6073 | 1.48 | SM (38:1) | C_43_H_87_N_2_O_6_P | [M-CH3]- |
| 769.6250 | 769.6229 | 1.43 | SM (40:2) | C_45_H_89_N_2_O_6_P | [M-CH3]- |
| 771.6413 | 771.6386 | 1.43 | SM (40:1) | C_45_H_91_N_2_O_6_P | [M-CH3]- |
| 795.6391 | 795.6386 | 1.38 | SM (42:3) | C_47_H_91_N_2_O_6_P | [M-CH3]- |
| 797.6561 | 797.6542 | 1.38 | SM (42:2) | C_47_H_93_N_2_O_6_P | [M-CH3]- |
| 799.6731 | 799.6699 | 1.38 | SM (42:1) | C_47_H_95_N_2_O_6_P | [M-CH3]- |

**Supplementary Table S11**. Tentatively identified gangliosides.

| Exp. Mass | Exact Mass | Error (ppm) | Name | Formula | Adduct |
| --- | --- | --- | --- | --- | --- |
| 1179.7417 | 1179.7372 | 3.81 | GM3 (36:1) | C_59_H_108_N_2_O_21_ | [M-H]- |
| 1235.8027 | 1235.7998 | 3.64 | GM3 (40:1) | C_63_H_116_N_2_O_21_ | [M-H]- |
| 1261.8196 | 1261.8154 | 3.57 | GM3 (42:2) | C_65_H_118_N_2_O_21_ | [M-H]- |
| 1354.7841 | 1354.7853 | 3.32 | GM2 (34:1) | C_65_H_117_N_3_O_26_ | [M-H]- |
| 1382.8215 | 1382.8166 | 3.94 | GM2 (36:1) | C_67_H_121_N_3_O_26_ | [M-H]- |
| 1426.8463 | 1426.8428 | 3.15 | GM2 (38:1) | C_69_H_125_N_3_O_27_ | [M-H]- |
| 1466.9136 | 1466.9105 | 3.07 | GM2 (42:1) | C_73_H_133_N_3_O_26_ | [M-H]- |
| 1470.8304 | 1470.8326 | 3.06 | GD3 (36:1) | C_70_H_125_N3O_29_ | [M-H]- |
| 1492.8176 | 1492.814 | 2.41 | GD3 (36:1) | C_70_H_125_N_3_O_29_ | [M+Na-2H]- |
| 1544.8724 | 1544.8694 | 2.91 | GM1 (36:1) | C_73_H_131_N_3_O_31_ | [M-H]- |
| 1572.9017 | 1572.9007 | 2.86 | GM1 (38:1) | C_75_H_135_N_3_O_31_ | [M-H]- |

**Supplementary Table S12**. Tentatively identified sulfatides.

| **Exp. Mass** | **Exact Mass** | **Error (ppm)** | **Name** | **Formula** | **Adduct** |
| --- | --- | --- | --- | --- | --- |
| 806.5451 | 806.5458 | 2.85 | SHexCer (36:1) | C_42_H_81_NO_11_S | [M-H]- |
| 834.5770 | 834.5771 | 2.76 | SHexCer (38:1) | C_44_H_85_NO_11_S | [M-H]- |
| 860.5912 | 860.5927 | 2.67 | SHexCer (40:2) | C_46_H_87_NO_11_S | [M-H]- |
| 862.6095 | 862.6084 | 2.67 | SHexCer (40:1) | C_46_H_89_NO_11_S | [M-H]- |
| 864.6227 | 864.6240 | 2.66 | SHexCer (40:0) | C_46_H_91_NO_11_S | [M-H]- |
| 884.5919 | 884.5927 | 2.60 | SHexCer (42:4) | C_48_H_87_NO_11_S | [M-H]- |
| 886.6073 | 886.6084 | 2.59 | SHexCer (42:3) | C_48_H_89_NO_11_S | [M-H]- |
| 888.6243 | 888.6240 | 2.59 | SHexCer (42:2) | C_48_H_91_NO_11_S | [M-H]- |
| 890.6403 | 890.6397 | 2.58 | SHexCer (42:1) | C_48_H_93_NO_11_S | [M-H]- |
| 892.6525 | 892.6553 | 2.58 | SHexCer (42:0) | C_48_H_95_NO_11_S | [M-H]- |
| 910.6097 | 910.6084 | 2.53 | SHexCer (44:5) | C_50_H_89_NO_11_S | [M-H]- |
| 912.6279 | 912.6240 | 2.52 | SHexCer (44:4) | C_50_H_91_NO_11_S | [M-H]- |
| 914.6379 | 914.6397 | 2.51 | SHexCer (44:3) | C_50_H_93_NO_11_S | [M-H]- |
| 916.6595 | 916.6553 | 2.51 | SHexCer (44:2) | C_50_H_95_NO_11_S | [M-H]- |
| 918.6709 | 918.6710 | 2.50 | SHexCer (44:1) | C_50_H_97_NO_11_S | [M-H]- |
| 936.6278 | 936.6240 | 2.46 | SHexCer (46:6) | C_52_H_91_NO_11_S | [M-H]- |
| 938.6424 | 938.6397 | 2.45 | SHexCer (46:5) | C_52_H_93_NO_11_S | [M-H]- |
| 964.6601 | 964.6553 | 2.38 | SHexCer (48:6) | C_54_H_95_NO_11_S | [M-H]- |
| 966.6732 | 966.6710 | 2.38 | SHexCer (48:5) | C_54_H_97_NO_11_S | [M-H]- |

**Supplementary Table S13.** List of discriminatory lipids identified from the AUC-ROC analysis comparing high tumor cell regions (HTC) to low-moderate tumor cell regions. HTC regions from N167 included GD3^High^, GD3^Low^, and palisading tumor cells (Pal.), while HTC regions from N118 and N158 contained mixed tumor cell populations. Calculated *p*-values measured by t-test are all <0.001.
